# Conformational surveillance of Orai1 by a rhomboid intramembrane protease prevents inappropriate CRAC channel activation

**DOI:** 10.1016/j.molcel.2021.10.025

**Published:** 2021-12-02

**Authors:** Adam G. Grieve, Yi-Chun Yeh, Yu-Fen Chang, Hsin-Yi Huang, Lucrezia Zarcone, Johannes Breuning, Nicholas Johnson, Kvido Stříšovský, Marion H. Brown, Anant B. Parekh, Matthew Freeman

**Affiliations:** 1 Sir William Dunn School of Pathology, University of Oxford, Oxford, OX1 3RE, UK; 2 Department of Physiology, Anatomy and Genetics, University of Oxford, Oxford, OX1 3PT, UK; 3 LumiSTAR Biotechnology, Inc., National Biotechnology Research Park, Taipei City 115, Taiwan; 4 Institute of Organic Chemistry and Biochemistry of the Czech Academy of Sciences (IOCB), Prague, 166 10, Czech Republic

**Keywords:** rhomboid protease, CRAC channel, Orai1, RHBDL2, transmembrane, calcium, T cell, signalling

## Abstract

Calcium influx through plasma membrane calcium release-activated calcium (CRAC) channels, which are formed of hexamers of Orai1, is a potent trigger for many important biological processes, most notably in T cell-mediated immunity. Through a bioinformatics-led cell biological screen, we have identified Orai1 as a substrate for the rhomboid intramembrane protease RHBDL2. We show that RHBDL2 prevents stochastic calcium signaling in unstimulated cells through conformational surveillance and cleavage of inappropriately activated Orai1. A conserved disease-linked proline residue is responsible for RHBDL2’s recognizing the active conformation of Orai1, which is required to sharpen switch-like signaling triggered by store-operated calcium entry. Loss of RHBDL2 control of CRAC channel activity causes severe dysregulation of downstream CRAC channel effectors, including transcription factor activation, inflammatory cytokine expression, and T cell activation. We propose that this surveillance function may represent an ancient activity of rhomboid proteases in degrading unwanted signaling proteins.

## Highlights

• A screen for transmembrane substrates of the rhomboid intramembrane protease RHBDL2
• RHBDL2 cleaves the CRAC channel protein Orai1 when it is inappropriately activated
• Conformational change in these calcium channels is recognized by RHBDL2
• An Orai1 mutant that cannot be cleaved by RHBDL2 causes a human disease syndrome

## Graphical abstract

**Figure:**
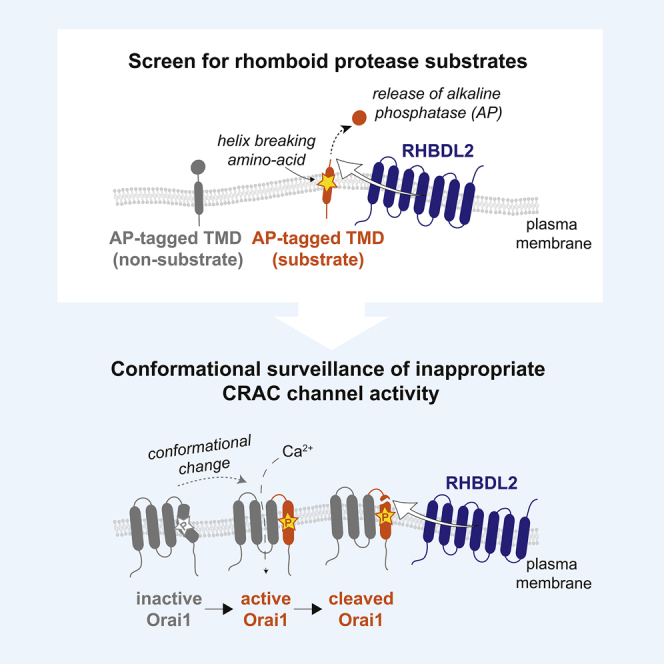

## Teaser

Rhomboids are conserved intramembrane proteases, but their roles in mammals are unclear because we do not know their true substrates. Grieve et al. show that RHBDL2 cleaves the calcium channel protein Orai1, recognizing conformational changes and thereby preventing inappropriate immunological signaling. A disease-causing Orai1 mutation renders this conformational surveillance ineffective.

## Introduction

Signaling controls most cellular functions and must therefore be precisely regulated in time and space. Although some signals produce graded responses, most are converted into binary outputs: having received an input, usually a ligand binding to a receptor, a cell changes its state in a switch-like way. Much signaling is triggered by integral membrane receptors, including, for example, growth factor and cytokine receptors, ion channels, and G protein-coupled receptors (GPCRs). To maintain switch-like signaling, it is essential that spontaneous activation is prevented in the absence of stimulus, but the post-translational control mechanisms for surveillance and prevention of inappropriate signaling are largely unknown. More generally, little is known about the regulation of the abundance and activity of most cell surface signaling proteins.

A good example of switch-like signaling is the control of calcium ion (Ca^2+^) flux across the eukaryotic plasma membrane (PM), which acts as a barrier between high extracellular and low cytoplasmic Ca^2+^ concentrations. Almost all cells in the animal kingdom regulate the level of cytosolic Ca^2+^ to control their function: a sharp rise in cytosolic Ca^2+^ controls enzymatic activity, protein-protein interactions, gene activation, cell proliferation, and apoptosis (Berridge et al., 2000). One of the primary routes of regulating Ca^2+^ entry in non-excitable cells is via Ca^2+^ release-activated Ca^2+^ (CRAC) channels (Parekh and Putney, 2005). Opening of CRAC channels at the cell surface causes a rapid increase of cytosolic Ca^2+^, which activates important signaling pathways, the most studied being in the adaptive immune system. The pore-forming subunits of CRAC channels are Orai proteins (Feske et al., 2006; Prakriya et al., 2006; Vig et al., 2006; Zhang et al., 2006). Orai1, the founding and most ubiquitously expressed member of the family, was originally identified as a genetic cause of severe combined immunodeficiency in humans and plays essential roles in T cell immunity (Feske et al., 2006).

CRAC channel activity must be transient and tightly controlled to prevent aberrant signaling. Indeed, leaky or defective CRAC channel activity is a direct cause of a group of diseases collectively referred to as channelopathies (Feske, 2010). CRAC channels are gated by the store-operated Ca^2+^ entry (SOCE) signaling pathway, in response to the stimulated release of stored endoplasmic reticulum (ER) Ca^2+^ (Hogan and Rao, 2015; Prakriya and Lewis, 2015). Reduction of ER Ca^2+^ is sensed by the ER-resident membrane proteins Stim1/2 (Hogan and Rao, 2015), and their interaction with Orai1 at PM-ER contact sites leads to CRAC channel opening and the influx of Ca^2+^. An emerging model states that Stim2, which senses more subtle changes in ER Ca^2+^ than Stim1, is pre-loaded at PM-ER contact sites and promotes the Stim1-Orai1 interaction upon mild or incomplete Ca^2^ store depletion (Burdakov and Verkhratsky, 2006; Brandman et al., 2007; Subedi et al., 2018). Channel opening relies both on Orai1 multimerization into a pore-forming hexameric unit and a concerted set of conformational changes in the transmembrane domains (TMDs) of Orai1 (Hou et al., 2012, 2018; Cai et al., 2016). The main interaction with the cytoplasmic domain of Stim1/2 occurs via the C-terminal cytoplasmic domain of Orai1, which is anchored to the membrane by its fourth TMD (Park et al., 2009). This interaction triggers allosteric activation and opening of CRAC channels (Yeung et al., 2020). Importantly, the correct stoichiometry between Orai1 and Stim1/2 is essential for normal SOCE (Mercer et al., 2006; Peinelt et al., 2006; Soboloff et al., 2006; Scrimgeour et al., 2009; Hoover and Lewis, 2011; Yeh et al., 2020). Overall, Stim1 binding and trapping of Orai1 at PM-ER contact sites is the rate-limiting step in CRAC channel activation and is therefore a major regulatory switch for Ca^2+^ influx and downstream signaling.

One class of enzymes that has the capacity to be involved in regulating the signaling function of integral membrane proteins by inactivation or degradation are intramembrane proteases, which use their active sites in the lipid bilayer to cleave TMDs of substrates. Most known functions of intramembrane proteases are to release signaling domains from membrane-tethered precursors, thereby triggering a signaling event. However, a wider range of roles is becoming apparent, including participating in some forms of ER-associated degradation (Fleig et al., 2012). Rhomboids are evolutionarily widespread intramembrane serine proteases. Despite extensive study and well-understood functions in several species (Freeman, 2014), and some scattered knowledge of their mammalian function (Lohi et al., 2004; Adrain et al., 2011; Fleig et al., 2012; Johnson et al., 2017), a comprehensive understanding of their physiological importance in mammals has been hampered by a lack of validated substrates (Lastun et al., 2016). To date, most identified rhomboid substrates are type I, single-pass transmembrane proteins. One feature that appears to be common to many intramembrane protease substrates is helical instability in transmembrane segments (Ye et al., 2000; Lemberg and Martoglio, 2002; Urban and Freeman, 2003), often created by the presence of helix-breaking residues such as prolines or glycines. The importance of this feature to rhomboid recognition is highlighted not only by their conservation and functional necessity but also by the observation that otherwise uncleavable TMDs can be converted into rhomboid substrates by the introduction of a proline residue (Moin and Urban, 2012). This raises the possibility that rhomboids may have originally evolved to recognize non-canonical transmembrane helices.

Our overall goal is to discover the conceptual and mechanistic themes associated with rhomboid intramembrane proteolysis and to uncover their physiological roles in mammals. Here, through a bioinformatics-led cell biological screen, we identify the fourth TMD of Orai1 as a substrate for the PM-localized rhomboid protease RHBDL2. We show that proteolysis of Orai1 by RHBDL2 sharpens the precision of SOCE by preventing stimulus-independent CRAC channel activation and inflammatory cytokine expression. Mechanistically, RHBDL2 prevents this inappropriate signaling through conformational selection and cleavage of the activated form of Orai1. The pathophysiological importance of this mechanism is highlighted by our demonstration that an activating disease-associated proline-to-leucine mutation in Orai1 TMD4 impairs RHBDL2 recognition, and severe defects in primary T cell activation occur upon RHBDL2 loss. We propose that conformational surveillance of polytopic proteins may represent an ancient rhomboid protease activity that could predate its better known roles in the cleavage and extracellular release of signaling molecules.

## Results

### Orai1 is an RHBDL2 substrate

To discover substrates for the rhomboid intramembrane protease, RHBDL2, we used a bioinformatic approach, followed by functional validation with a cell-based assay. We focused on three characteristics of known rhomboid substrates: their TMDs have a type I orientation (NH_2_-out, COOH-in), many contain extracellular EGF-like domains, and they often contain helix-destabilizing amino acids (Freeman, 2014). Therefore, we identified candidates by the presence of extracellular EGF domains and/or through profile-profile alignments using the online server HHpred (Zimmermann et al., 2018) to find type I TMDs that have structural similarity to one of the best characterized rhomboid substrates, *Drosophila* Spitz (Freeman, 2014).

**Figure 1:**
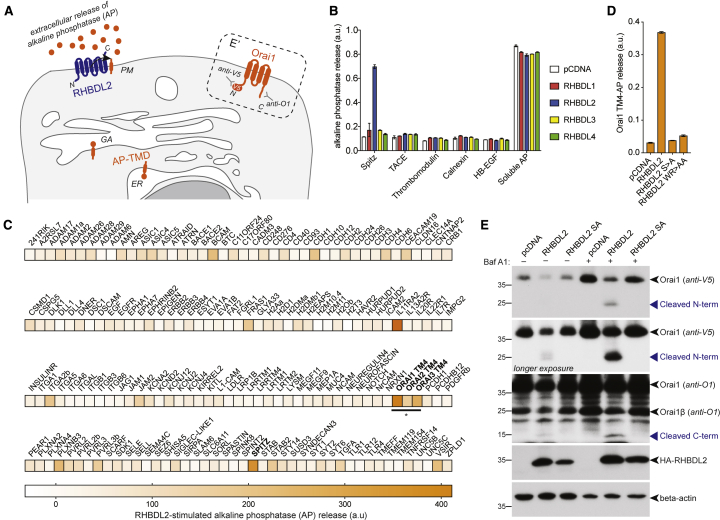
Orai1 is an RHBDL2 substrate (A) Scheme of the alkaline phosphatase-transmembrane domain (AP-TMD) screen. Each AP-TMD (orange) has a signal sequence for ER insertion. Upon co-expression with RHBDL2 (blue), if AP-TMD is cleaved, it will release AP into the extracellular medium. In the dashed box (E′), the topology of V5-Orai1 is illustrated, indicating the epitopes recognized by antibodies in (E). GA, Golgi apparatus; PM, plasma membrane. For the screen, mouse sequences for RHBDL2 and the candidate TMDs were used. (B–D) HEK293 cells were transfected with pcDNA, 3xHA-RHBDL1-4, or RHBDL2 mutants (S → A or WR → AA) and indicated AP-TMDs for 48 h. Soluble AP is AP with a signal sequence but no TMD. Released AP was collected over the final 16 h of expression. Values represent the level of released AP/total AP (B and D); in (C) these values were converted into a normalized level of “RHBDL2-stimulated AP release” (for each AP-TMD: AP release upon RHBDL2 expression was divided by values taken for pcDNA transfected controls multiplied by 100). All values have AP release with pcDNA subtracted (which is RHBDL2 independent). For the screen, n = 2 biological repeats for each AP-TMD. Error bars indicate SEM. (E) Western blots of lysates from HEK293 cells transfected with V5-hOrai1 and indicated RHBDL2 constructs for 48 h, treated with 100 nM bafilomycin A1 (Baf A1) for 16 h and probed with indicated antibodies. Different full-length forms of Orai1 (Orai1β arises from alternative methionines; Fukushima et al., 2012); cleavage products are indicated by blue arrowheads.

The top bioinformatic TMD hits (Table S1) were inserted into a reporter that was co-expressed with RHBDL2, so that RHBDL2-dependent cleavage led to accumulation of extracellular alkaline phosphatase (AP) (Figure 1A, left), which was detected using a colorimetric phosphatase assay. As a positive control, we used the TMD of Spitz, which can be cleaved by RHBDL2 (Figures 1B and S1A) (Urban and Freeman, 2003; Strisovsky et al., 2009). Encouragingly, previously identified substrates such as BCAM, cadherins, and Ephrin proteins were among the top hits (Johnson et al., 2017; Battistini et al., 2019); the top hit in our screen, IL11RA2, was also recently reported as an RHBDL2 substrate (Koch et al., 2021). Among our strongest validated hits, we found the fourth TMD of all three members of the Orai family of Ca^2+^ channels (Figures 1A and 1C). The cleavage required the catalytic serine residue of RHBDL2, as well as the hallmark rhomboid WR motif in the L1 loop that connects TMD1 and TMD2 (Figure 1D) (Lemberg and Freeman, 2007).

We tested whether RHBDL2 could cleave full-length Orai1, which unlike most known rhomboid substrates is a polytopic protein (Figure 1A, E′). Upon expression with RHBDL2, full-length Orai1 and its short isoform (Orai1β) were cleaved into two fragments of molecular weights that confirmed cleavage within the fourth TMD (Figure 1E). Immunofluorescent labeling showed that cleavage led to Orai1 internalization from the PM, suggesting that it was degraded as a consequence of cleavage (Figure S1B). Accordingly, treatment of cells with bafilomycin A1, a lysosomal degradation inhibitor, increased the levels of the N- and C-terminal Orai1 cleavage products (Figure 1E). Rhomboid substrates are normally cleaved between amino acids with small side chains, and bulky residues at the cleavage site can often render them uncleavable (Urban and Freeman, 2003). HHpred-generated alignments between TMD4 of Orai1 and the Spitz TMD showed perfect alignment of the alanine-serine cleavage site in Spitz with alanine-241/serine-242 in mouse Orai1 (Strisovsky et al., 2009) (Figure S1C). Consistent with the expectation that they constitute the site of Orai1 cleavage, both A241F and S242F mutations blocked RHBDL2-dependent proteolysis of Orai1 TMD4, as did mutation to phenylalanine of the nine extracellular amino acids (F9) of TMD4 (Figures S1D and S1E). Overall, these results confirm that the fourth TMD of Orai1 is a bona fide substrate of RHBDL2 and that cleavage triggers subsequent Orai1 degradation in lysosomes.

### RHBDL2 controls CRAC channel activity

**Figure 2:**
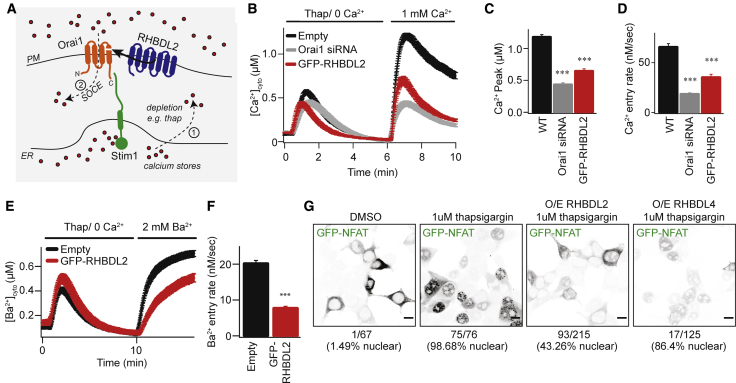
RHBDL2 downregulates CRAC channel activity (A) Overview of the SOCE pathway. Upon depletion of ER Ca^2+^ stores (red dots) by thapsigargin (step 1), Stim1 (green) oligomerizes and extends into ER-PM contact sites. It traps and nucleates Orai1 (orange) into functional CRAC channels (step 2). For simplicity, Stim1 and Orai1 are illustrated as monomeric. RHBDL2 (blue) cleaves the fourth TMD in Orai1, which anchors the C-terminal Stim1 interaction site. (B) SOCE is monitored by cytosolic Fura-2 fluorescence and compared between control HEK293 cells and those transfected with GFP-RHBDL2 or Orai1 siRNA. Cells were stimulated with 2 μM thapsigargin in Ca^2+^-free buffer, followed by readmission of 1 mM external Ca^2+^. (C and D) Aggregate data from cells treated as in (B) are plotted, analyzing the peak Ca^2+^ level in each condition (C) and rate of Ca^2+^ entry (D). Each bar in (C) and (D) represents between 34 and 68 cells. (E) Ba^2+^ entry is compared between cells transfected with empty vector or GFP-RHBDL2, after treatment with 2 μM thapsigargin in Ba^2+^/Ca^2+^-free buffer. (F) The rate of Ba^2+^ entry is plotted; each bar represents between 12 and 19 cells. For two-tailed t tests, ^∗∗∗^p < 0.001. In bar charts, error bars indicate SEM. (G) PFA-fixed HEK293 cells transfected with NFAT1(1-460)-GFP and indicated RHBDL2/4 constructs were treated with DMSO or 1 μM thapsigargin for 45 min. Single confocal sections of GFP fluorescence are depicted with inverted grayscale lookup tables. Scale bar, 10 μm.

The fourth TMD of Orai1, which we have shown to be an RHBDL2 substrate, both anchors the cytoplasmic C-terminal Stim-interacting domain and is proposed to be central to the conformational changes that initiate CRAC channel opening (Park et al., 2009; Yeung et al., 2020) (Figure 2A). We therefore tested the effect of RHBDL2 expression on endogenous SOCE by monitoring cytosolic Ca^2+^ with the reporter dye Fura-2. Signaling was triggered by thapsigargin treatment, which depletes ER Ca^2+^, followed by addition of physiological levels of extracellular Ca^2+^. We found that RHBDL2 expression reduced SOCE by ∼50%, a level similar to that observed upon Orai1 depletion by small interfering RNA (siRNA) (Figures 2B–2D). In these standard assays, cytosolic Ca^2+^ reflects a balance of Ca^2+^ influx and efflux, thus making it formally possible that the observed difference was not the direct result of altered CRAC channel activity but instead accelerated Ca^2+^ efflux by PM Ca^2+^-ATPases. To discriminate between these two scenarios, we assayed influx of barium ions (Ba^2+^), which can be transported through CRAC channels but cannot be pumped out of the cell by PM Ca^2+^-ATPases (Hoth, 1995; Bakowski and Parekh, 2007). Again, there was a ∼50% diminished influx of Ba^2+^ after thapsigargin treatment, supporting the conclusion that RHBDL2 expression prevented CRAC channel activity directly (Figures 2E and 2F). To investigate the functional correlate of this effect, we examined the nuclear translocation of the transcription factor NFAT (nuclear factor of activated T cells), which is triggered by CRAC channel activity (Hogan et al., 2010; Kar et al., 2011; Kar and Parekh, 2013). Expression of RHBDL2, but not the related rhomboid protease RHBDL4, inhibited GFP-NFAT nuclear translocation upon treatment with thapsigargin (from ∼98% nuclear NFAT upon thapsigargin treatment in control cells to ∼43% nuclear NFAT in RHBDL2-expressing cells) (Figure 2G). We conclude that RHBDL2 cleavage of Orai1 prevents both endogenous CRAC channel activity and the stimulated nuclear translocation of NFAT.

**Figure 3:**
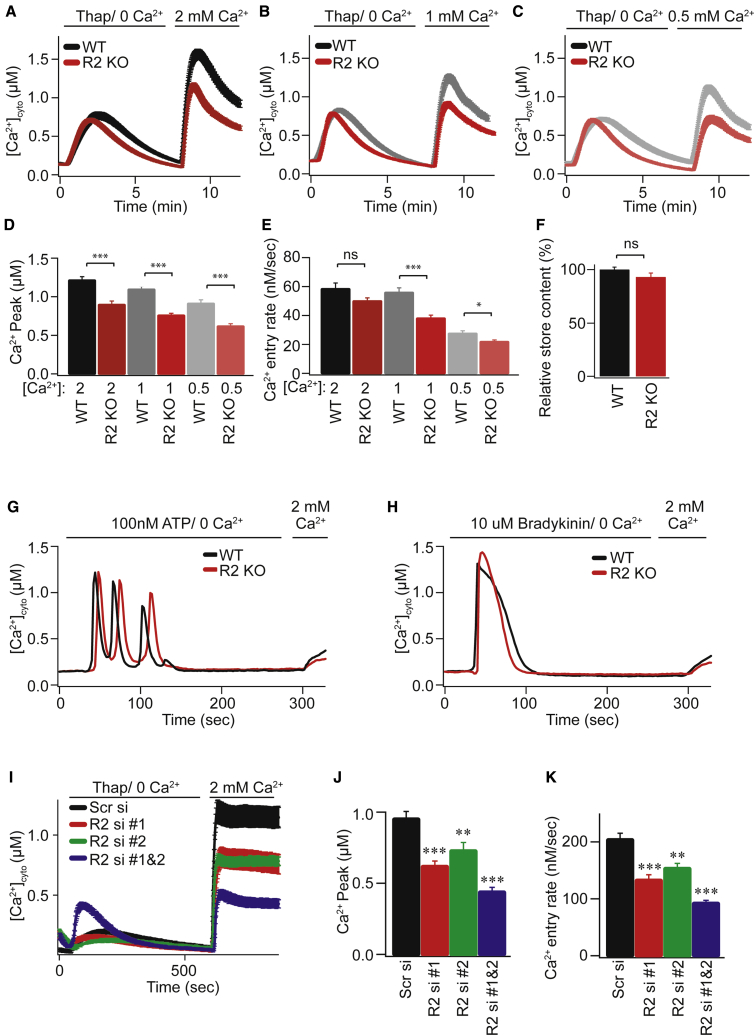
RHBDL2 regulates normal SOCE (A–C) SOCE is monitored by cytosolic Fura-2 fluorescence and compared between wild-type and RHBDL2 mutant HEK293 cells (CRISPR-mediated deletion of the catalytic histidine; R2 KO). Cells were stimulated with 2 μM thapsigargin in Ca^2+^-free buffer, followed by readmission of external Ca^2+^. (D–F) Aggregate data from cells treated as in (A)–(C) are plotted, analyzing the peak Ca^2+^ level in each condition (D), rate of Ca^2+^ entry (E), and total ER Ca^2+^released by thapsigargin treatment (F). Each bar in (D)–(F) represents between 11 and 35 cells. (G–I) SOCE monitored as in (A)–(C), but with indicated doses of ATP or bradykinin in place of thapsigargin in (G) and (H), or comparing scrambled siRNA and RHBDL2 siRNA-treated HaCaT cells in (I). Cells were stimulated in Ca^2+^-free buffer, followed by readmission of 2 mM external Ca^2+^. (J and K) Aggregate data from cells treated as in (I) are plotted, analyzing the peak Ca^2+^ level in each condition (J) and rate of Ca^2+^ entry (K). Each bar in (J) and (K) represents between 22 and 36 cells. For two-tailed t tests, ^∗^p < 0.05, ^∗∗^p < 0.01, and ^∗∗∗^p < 0.001, compared with wild-type or scrambled siRNA controls. Error bars indicate SEM.

RHBDL2 expression inhibits CRAC channel signaling, but is cleavage of Orai1 a physiologically meaningful event? To address this, we tested whether loss of RHBDL2 function had a physiological effect on Ca^2+^ signaling and the outputs of SOCE. We first used HEK293 mutant cells, in which RHBDL2 was mutated by deleting the catalytic histidine by CRISPR-Cas9 editing (Figures S2A and S2B). These cells displayed reduced endogenous SOCE across a range of physiological extracellular Ca^2+^ concentrations (Figures 3A–3E). We observed no significant difference in the amount of Ca^2+^ released from stores by thapsigargin (Figure 3F), implying that there was no defect in the upstream pathway that triggers SOCE. Furthermore, defects in SOCE were also observed when physiological activators of phospholipase C, such as ATP and bradykinin (Figures 3G and 3H; quantification in Figures S3A and S3B) were used to trigger SOCE. Knockout (KO) cells responded indistinguishably from wild-type (WT) cells when treated with the Ca^2+^ ionophore, ionomycin, indicating no general defects in Ca^2+^ signaling (Figure S3C). Together these results demonstrate that loss of RHBDL2 in HEK cells causes defects in SOCE. We also assayed the effect of RHBDL2 depletion in HaCaT keratinocytes (Figure S3D). Using two different siRNAs, there was a clear defect in SOCE when RHBDL2 was depleted (Figures 3I–3K), further demonstrating that RHBDL2 does indeed regulate endogenous CRAC channel activity.

### RHBDL2 regulates human T cell activation

**Figure 4:**
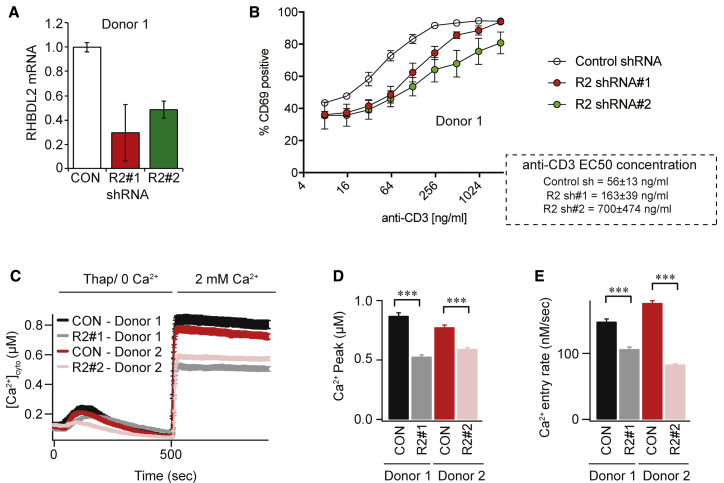
RHBDL2 regulates human T cell activation (A) TaqMan assays for RHBDL2 mRNA levels in T cells transduced with virus encoding control or RHBDL2 shRNAs. Error bars represent RQ standard error. (B) T cell activation measured by quantification of surface CD69 expression by fluorescence-activated cell sorting (FACS). CD69 expression is compared between control and RHBDL2 shRNA transduced primary CD4 T cells after stimulation with CD3. Each trace represents three biological replicates. In the dashed box, the calculated EC_50_ of anti-CD3 for each shRNA. Error indicates SEM. (C) SOCE is monitored by cytosolic Fura-2 fluorescence and compared between control and RHBDL2 shRNA transduced T cells. T cells were stimulated with 2 μM thapsigargin in Ca^2+^-free buffer, followed by readmission of 2 mM external Ca^2+^. (D and E) Aggregate data from T cells treated as in (C) are plotted, analyzing the peak Ca^2+^ level in each condition (D) and rate of Ca^2+^ entry (E). Each bar in (D) and (E) represents between 34 and 45 cells. For two-tailed t tests, ^∗∗∗^p < 0.001, compared with control shRNA transduced T cells. Error bars represent SEM.

CRAC channels participate in the primary activation of T cells by antigen-presenting cells and are thus centrally involved in T cell immunity (Feske, 2007). We therefore isolated primary CD4-positive T cells from two healthy human donors and asked whether RHBDL2 depletion (Figures 4A and S3E) affected their activation by anti-CD3 crosslinking of the T cell receptor, a widely used method of mimicking T cell interactions with antigen-presenting cells. Using surface CD69 expression as a readout (Ziegler et al., 1994), we found that T cell activation was reproducibly defective across three experiments (Figures 4B and S3F; half maximal effective concentration [EC_50_] values of anti-CD3 for each short hairpin RNA (shRNA) are indicated in the dashed box). We also directly measured CRAC channel activity in these RHBDL2-depleted T cells and found severely reduced SOCE (Figures 4C–4E). Combined, our results demonstrate not only that the role of RHBDL2 in controlling CRAC channel activity is essential for normal SOCE but also that this has a profound effect on human T cell activation.

### RHBDL2 controls signaling by optimizing stoichiometry between Orai1 and Stim1

**Figure 5:**
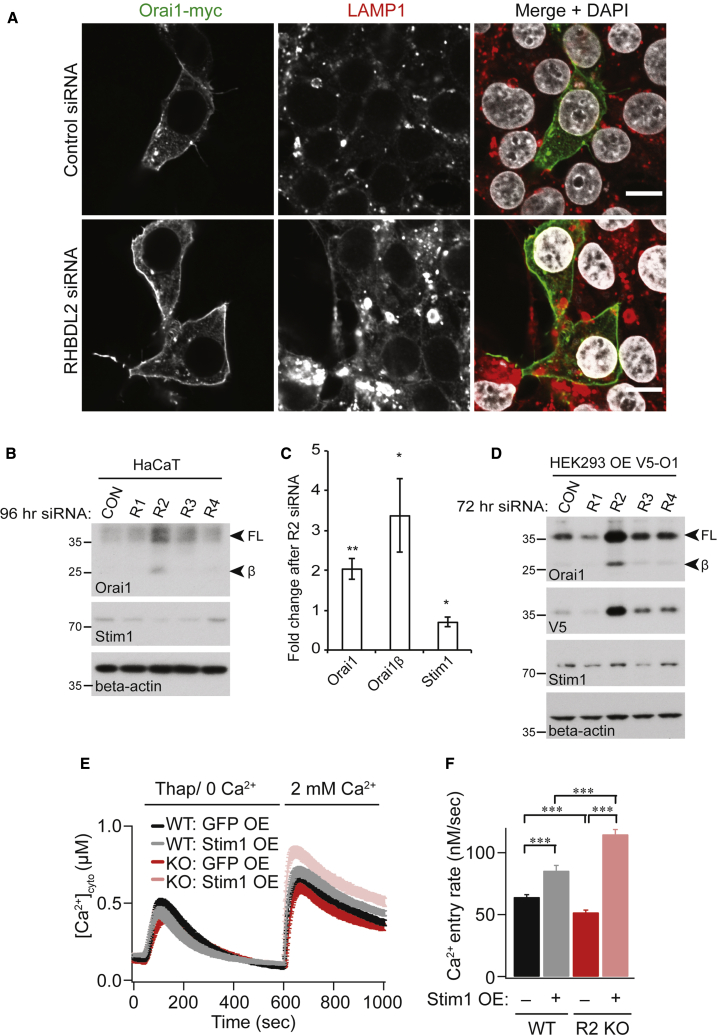
RHBDL2 controls signaling by optimizing stoichiometry between Orai1 and Stim1 (A) Immunofluorescent labeling of Orai1-myc and LAMP1 in HEK293 cells transfected with indicated siRNA for 72 h and transfected with hOrai1-myc 24 h prior to fixation. Scale bar, 10 μm. (B) Western blots of HaCaT lysates after cells were treated with control or RHBDL1-4 siRNAs for 96 h, labeled for endogenous Orai1, Stim1, and beta-actin. Full-length Orai1 (FL) and Orai1β (FLβ) are indicated by arrowheads. (C) Quantification of the fold change in Orai1 and Stim1 protein abundance, from three independent experiments performed as in (B). Error bars represent SEM. (D) Western blots of HEK293T lysates after cells were treated with control or RHBDL1-4 siRNAs for 72 h, expressing V5-hOrai1 for the final 24 h. Full-length Orai1 (FL) and Orai1β (FLβ) are indicated by arrowheads. (E) SOCE is compared between wild-type (WT) and RHBDL2 KO HEK293 cells overexpressing GFP or Stim1-YFP. Cells were stimulated with 2 μM thapsigargin in Ca^2+^-free buffer, followed by readmission of 2 mM external Ca^2+^. (F) Aggregate data from cells treated as in (E) are plotted, analyzing the rate of Ca^2+^ entry. Each bar in (F) represents between 29 and 46 cells. For two-tailed t tests, ^∗∗^p < 0.001 and ^∗∗∗^p < 0.001, compared with wild-type. Error bars represent SEM.

Superficially, the loss of RHBDL2 might be expected to lead to higher Orai1 levels, more CRAC channels, and therefore enhanced SOCE. We therefore sought to explain the counterintuitive result that loss of RHBDL2 led to decreased SOCE. We began by analyzing the subcellular localization of Orai1 in RHBDL2-depleted cells (Figure 5A). This experiment provided three important insights. First, Orai1 was still targeted to the PM. Second, Orai1 levels at the PM appeared elevated in RHBDL2-depleted cells. This was further confirmed by cell surface biotinylation experiments, which showed a specific elevation of PM Orai1 levels upon depletion of RHBDL2 (Figure S3G). Combined, this ruled out the possibility that the SOCE phenotype was due to a failure in trafficking of Orai1 to the PM. And third, we found that Orai1 did not accumulate in LAMP1-positive lysosomes, confirming that this increased pool of Orai1 at the PM upon knockdown of RHBDL2 was not a secondary consequence of defective lysosomal function. Consistent with its role in cleaving and targeting Orai1 for degradation, depletion of RHBDL2—but not other rhomboids RHBDL1, RHBDL3, or RHBDL4—led to an increase in endogenous full-length Orai1 and its shorter isoform, Orai1β (Figures 5B and 5C). Plasmid-borne Orai1 also accumulated specifically in RHBDL2-depleted cells, clearly demonstrating that transcriptional changes are not responsible for increased Orai1 protein (Figure 5D). Overall, these data indicate that RHBDL2 loss caused elevated levels of PM Orai1 but further prompted the question of how this led to decreased SOCE.

The answer to this question was provided by the fact that the correct stoichiometry between Orai1 and Stim1 is essential for the SOCE pathway (Figure 2A): Stim1 levels are rate limiting for CRAC channel activation, and excess Orai1 has a dominant-negative effect on SOCE (Mercer et al., 2006; Peinelt et al., 2006; Soboloff et al., 2006; Scrimgeour et al., 2009; Hoover and Lewis, 2011; Yeh et al., 2020). We found that RHBDL2 depletion caused PM Orai1 levels to increase, with a slight but reproducible decrease in Stim1 levels (Figures 5B–5D). We therefore predicted that if compromised stoichiometry was the cause of the observed SOCE defects, overexpression of Stim1 in RHBDL2 KO cells should rescue the phenotype by allowing production of functional CRAC channels and elevated SOCE. Accordingly, expression of Stim1 not only rescued the defect in SOCE but significantly enhanced its rate compared with WT cells expressing the same construct (Figures 5E and 5F). This result indicates that the defects in SOCE caused by RHBDL2 loss are caused by an imbalance in Orai1:Stim1 stoichiometry. An important implication of this result is worth emphasizing: the Orai1 that accumulates in the absence of RHBDL2 is functionally competent. This rules out another possible role for RHBDL2: that it might act in a misfolded protein quality control mechanism to degrade defective Orai1. Overall, these data show that RHBDL2 cleavage and subsequent degradation of Orai1 act to maintain an optimal stoichiometry between Orai1 and Stim1.

### RHBDL2 prevents inappropriate CRAC channel activation in resting cells

**Figure 6:**
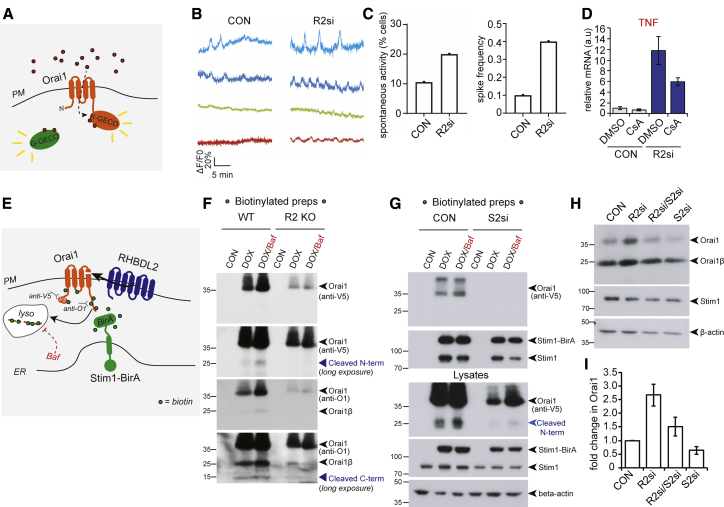
RHBDL2 prevents inappropriate CRAC channel activation in resting cells (A) Scheme of the hOrai1-K-GECO and Cyto-G-GECO reporter assay for spontaneous CRAC channel activity. K-GECO fluorescence is a measure of CRAC channel activity; G-GECO fluorescence is a measure of global cytoplasmic Ca^2+^ fluctuations. (B) Four representative line traces of K-GECO fluorescence in single HaCaT cells treated with control siRNA or RHBDL2 siRNA. Spontaneous CRAC channel activation is defined as a >20% increase in the peak amplitude (ΔF/F0). (C) Quantification of spontaneous CRAC channel activity, defined as a >20% increase above the baseline K-GECO fluorescence (see Figure S4C). Quantification of the percentage of cells displaying spontaneous CRAC channel activity (38 individual cells analyzed in control, 45 individual cells analyzed in RHBDL2 siRNA cells), and the K-GECO fluorescence spike frequency (number of spontaneous CRAC channel activity events over 10 min, relative to the number of cells). (D) TaqMan assay for TNF-alpha in HaCaT cells treated with control or RHBDL2 siRNAs for 48 h. Cyclosporin A (1 μm) was added for the final 24 h. Error bars represent RQ standard error. Each bar represents one of at least four biological replicates. (E) A scheme of the Stim1-BirA experiment in (F) and (G), illustrating the biotinylation of V5-hOrai1 (orange) by Stim1-BirA^∗^ (green) at PM-ER contact sites, and the consequence of RHBDL2 (blue) activity. Biotin is indicated by green dots. For simplicity, Stim1 and Orai1 are illustrated as monomeric. (F and G) Western blots of neutravidin agarose-based biotin preparations (in F and G, upper) and total cell lysates (in G, lower) from wild-type (WT) or RHBDL2 knockout (R2 KO) HaCaT cells. The expression of V5-hOrai1 and Stim1-BirA^∗^ was induced with doxycycline (DOX; 250 μg/mL final) for 96 h in the presence of 50 μm biotin. Six hours prior to lysis, bafilomycin A1 (BAF; 100 nm final) was added to block lysosomal degradation. In (G), cells were treated twice with Stim2 siRNAs for 96 h prior to lysis. Blots were probed for the N-terminal or C-terminal epitopes recognized by V5 and O1 antibodies, respectively. Stim1 and Stim1-BirA^∗^ were probed for using an anti-Stim1 antibody. Different full-length forms of Orai1 and their cleavage products are indicated by the blue arrowheads. (H) Western blots of HaCaT lysates after treatment with control and RHBDL2 siRNA for 72 h, and when used, Stim2 siRNA for 96 h, labeled for endogenous Orai1, Stim1, and beta-actin. Full-length Orai1 (FL) and Orai1β (FLβ) are indicated by arrowheads. (I) Quantification of the fold change in Orai1 protein abundance, from three independent experiments performed as in (H). Error bars represent SEM.

We next questioned the biological context of Orai1 cleavage by RHBDL2. We noted that PM Orai1 protein levels increased upon depletion of RHBDL2 even in unstimulated cells (Figures 5A and S3G), indicating that cleavage was not dependent on SOCE. We therefore hypothesized that the role of RHBDL2 cleavage of Orai1 is to prevent inappropriate CRAC channel activity in the absence of stimulation (i.e., that RHBDL2 maintains the baseline threshold level of CRAC channel signaling). To test this hypothesis, we developed a sensitive reporter assay for local CRAC channel activity: we fused the genetically encoded Ca^2+^ indicator K-GECO to Orai1 and measured its fluorescence at the PM over time (Figures 6A and S4A; Shen et al., 2018). We detected infrequent transient spikes in Orai1-K-GECO fluorescence in very few WT cells. Crucially, we found that these spikes in Orai1-K-GECO fluorescence sometimes correlated with increased cytosolic Ca^2+^ levels, measured by a co-expressed cytosolic reporter, cyto-G-GECO (Figure S4B, gray lines). This indicated that although the Orai1-K-GECO spikes were small and transient, this apparent CRAC channel activity was sufficient to transiently raise cytosolic Ca^2+^ levels. On the basis of these observations, we imposed a strict definition of spontaneous CRAC channel activity as a >20% increase in Orai1-K-GECO fluorescence, as this was the threshold at which we consistently observed concomitant increases in cyto-G-GECO fluorescence (Figure S4C). To further confirm that we were observing spontaneous CRAC channel activity, we performed the same measurements in the presence of the CRAC channel inhibitor, BTP2, or in the absence of extracellular Ca^2+^. Under these conditions, we observed neither spontaneous CRAC channel activation nor spikes in cytosolic Ca^2+^ (Figures S4D and S4E).

Having validated this assay, we tested the effect of RHBDL2 loss on spontaneous activation of CRAC channels at the PM (Figure 6B). Confirming our hypothesis, we found that not only did the number of cells displaying spontaneous activity increase in the absence of RHBDL2, but the frequency of such events also increased (Figures 6C and 6D). Overall, this approach provided a direct readout of the effect of RHBDL2 loss on CRAC channel activity, revealing that RHBDL2 acts to prevent stimulus-independent signaling. Using chemical crosslinking approaches we also found elevated levels of higher molecular weight Orai1-positive complexes upon RHBDL2 loss, in line with a small number of Orai1 molecules assembling into multimeric CRAC channels (Figures S5A and S5B).

Do these low-level transient events have any meaningful effect on downstream signaling pathways? As NFAT nuclear translocation has been reported to be highly sensitive to low-level local CRAC channel activation (Kar et al., 2011; Kar and Parekh, 2013), we decided to follow NFAT-dependent gene expression as a further readout of CRAC channel activation. Strikingly, we found that RHBDL2 depletion in HaCaT cells led to a 19-fold upregulation of the expression of the inflammatory cytokine TNF, one of the major NFAT responsive genes (Rao et al., 1997) (Figure S5C; 72 h siRNA). Cytokines not dependent on NFAT, such as IL-6, were not affected (Figure S5C). We confirmed that the elevated TNF expression was indeed due to upregulated NFAT, as it was inhibited by treatment with cyclosporin A, a widely used inhibitor of NFAT signaling (Rao et al., 1997) (Figures 6D and S5D). Together, these data demonstrate that RHBDL2 is needed to prevent inappropriate CRAC channel activity and its downstream signaling pathways, such as NFAT-dependent transcription.

We next questioned the molecular mechanisms that underpinned this excess signaling, hypothesizing that it was caused by stochastic, stimulus-independent Stim1 activation of CRAC channels. This was prompted by two observations. First, PM-ER contact sites stably exist regardless of Stim1 activation (Wu et al., 2006; Orci et al., 2009), and Stim1 targeting to PM-ER contact sites is Orai1 independent (Liou et al., 2007; Park et al., 2009). Second, Stim2, which is prelocalized at PM-ER contact sites, can promote Stim1 translocation in conditions of incomplete depletion of ER Ca^2+^ stores (Burdakov and Verkhratsky, 2006; Brandman et al., 2007; Subedi et al., 2018). We therefore hypothesized that the unwanted CRAC channel activity against which RHBDL2 protects cells may be stochastic, triggered by random Stim1/Orai1 interaction in the absence of stimulation.

To test this idea, we developed an assay to identify Orai1 molecules that have previously encountered Stim1, by fusing the BirA^∗^ biotin ligase to the cytoplasmic domain of Stim1. We then asked whether the subset of Orai1 molecules that had encountered Stim1, and were therefore biotinylated, were preferentially cleaved by RHBDL2 (Figure 6E). At rest, in unstimulated WT cells, a small proportion of Orai1 (0.38% ± 0.13% of the total pool after 72 h of expression) does indeed encounter Stim1 (Figure 6F, DOX; Figure S5E for Stim1). Importantly, this pool of Orai1 was cleaved in WT cells in an RHBDL2-dependent manner (Figure 6F, N- and C-terminal cleavage products in WT versus KO; Figures S2C and S2D). Blocking lysosomal degradation increased these cleavage products. The central message of this experiment is that Orai1 was cleaved by endogenous RHBDL2, and subsequently degraded in lysosomes, after stimulus-independent engagement with Stim1.

Although this experiment indicates that RHBDL2 cleaves Orai1 that has encountered Stim1, we cannot definitively rule out Orai1 cleavage in other contexts. Upon initial generation of these cell lines, Orai1 cleavage products were strictly limited to the biotinylated pool and never detected in total lysates (Figure S5F; corresponding lysates for Figure 6F). However, after long-term passage, RHBDL2-dependent Orai1 cleavage products became detectable in lysates (Figures 6G and S5H), suggesting that in those cells the level of cleavage occurring was greater than just the biotinylated pool. We do not know what causes this change in behavior as the cell line passage number increases, but it does not challenge the primary conclusion that RHBDL2 cleaves Orai1 that has encountered Stim1 at ER-PM contact sites.

Providing a mechanistic understanding of how this low-level PM-ER interaction occurs, we found that Stim1-dependent Orai1 biotinylation was dependent on Stim2 levels (Figures 6G and S5G). Consistent with its role in this pathway, Stim2 loss led to a strong decrease in Orai1 cleavage (Figure S5H), and prevented the upregulation of endogenous Orai1 levels upon loss of RHBDL2 (Figures 6H and 6I). This reinforces the evidence that RHBDL2 primarily cleaves forms of Orai1 that have engaged Stim1. Furthermore, these highlight the critical role that Stim2 plays in regulation of the Orai1-Stim1 interaction at PM-ER contact sites in unstimulated cells.

Overall, these results define a central conclusion of our work: that RHBDL2 promotes the cleavage and subsequent lysosomal degradation of Orai1 molecules that have previously encountered Stim1/2. In unstimulated cells, this population of molecules is small, but, as shown in Figures 4B, 6B–6D, and S5C, they nevertheless trigger a dangerous level of unwanted Ca^2+^ signaling if allowed to accumulate. Without RHBDL2 acting as a brake on this stochastic CRAC channel activity, T cell activation and inflammatory cytokine expression, for example, are both severely defective.

### RHBDL2 recognition of Orai1 is sensitive to calcium and conformationally determined

**Figure 7:**
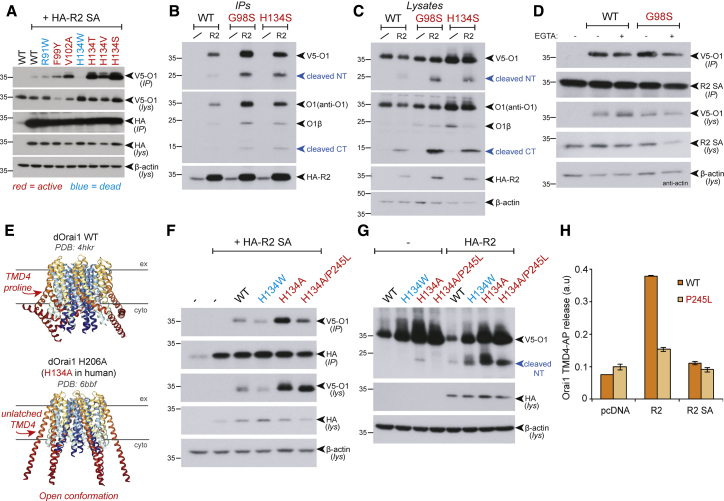
RHBDL2 recognition of Orai1 is sensitive to calcium and conformationally determined (A–D) HA immunoprecipitates (IP) and inputs from HEK293 cells transiently expressing WT or SA mutant 3xHA-RHBDL2 and wild-type or mutant V5-hOrai for 24 h were immunoblotted for V5, HA, and beta-actin. In (D), 5 min prior to lysis, cells were washed in PBS and incubated with external solution containing 2 mM Ca^2+^ or Ca^2+^-free solution containing 1 mM EGTA. This panel is representative of at least three separate repeat results for each Orai1 construct. (E) Structures of *Drosophila* Orai WT (left) or Orai H206A (right), highlighting the accessibility of the fourth TMD and the large change in conformation around the *Drosophila* equivalent of proline-245 in human Orai1. (F) HA immunoprecipitates (IP) and inputs from HEK293 cells transiently expressing 3xHA-RHBDL2 SA and wild-type or mutant V5-hOrai for 24 h were immunoblotted for V5, HA, and beta-actin. (G) Lysates from HEK293 cells transiently expressing pcDNA3.1 or WT 3xHA-RHBDL2 and wild-type or mutant forms of V5-hOrai for 24 h were immunoblotted for V5, HA, and beta-actin. (H) HEK293 cells were transfected with pcDNA, 3xHA-RHBDL2 or RHBDL2 SA, and AP-TMD4 or AP-TMD P245L from hOrai1 for 48 h. Released AP was collected over the final 16 h. Values represent the level of released alkaline phosphatase/total alkaline phosphatase. Error bars represent SEM.

The proposed model of RHBDL2 patrolling the PM to destroy inappropriately active CRAC channels suggests that the protease may preferentially recognize specific forms of Orai1, engaged by Stim1. Such conformational selectivity of rhomboid proteases has not previously been reported. To address the idea, we capitalized on elegant structure-function data that provide a detailed understanding of the contributions of specific Orai1 TMD amino acids to overall CRAC channel architecture and activity (Yamashita et al., 2017; Hou et al., 2018). Mutation of histidine-134 in Orai1 to alanine, threonine, valine, or serine constitutively activates Orai1 (Yeung et al., 2018), as do other mutations such as F99Y, V102A, and P245L (Nesin et al., 2014; Palty et al., 2015). Conversely, other Orai1 TMD mutations have a profound inactivating effect (G98C [Yamashita et al., 2017], R91W [Feske et al., 2006], and H134W [Yeung et al., 2018]). We compared binding of RHBDL2-SA (the serine-to-alanine catalytic mutant, which binds stably to substrates) to these different Orai1 activity mutants. There was a clear correlation between RHBDL2-SA binding and Orai1 activity: RHBDL2 bound strongly to Orai1 H134S, the mutant that is closest in its properties to a Stim1-gated CRAC channel (Yeung et al., 2018). In contrast, inactive mutants of Orai1, such as H134W, showed very weak binding to RHBDL2 (Figures 7A, 7B, and S6A). When performing the same experiments with active RHBDL2 we observed a similar binding preference for active forms of Orai1 (Figure 7B) and found increased levels of Orai1 cleavage products (Figure 7C). Overall, this demonstrated that RHBDL2 exhibits selectivity for active forms of Orai1.

Among the active mutants of Orai1 that RHBDL2 preferentially bound was G98S (Figures 7B and S6B), in which there is unregulated opening of the channel pore and influx of Ca^2+^. One of the *Drosophila* rhomboids is activated by Ca^2+^(Baker and Urban, 2015), so we tested whether RHBDL2 activity itself was stimulated by elevated cytosolic Ca^2+^. Thapsigargin treatment did not increase cleavage of Orai1 TMD4 (Figure S6D), indicating that RHBDL2 is not catalytically activated by increased cytosolic Ca^2+^ or depletion of ER Ca^2+^ stores. We did, however, find that RHBDL2 recognition of full-length Orai1 is sensitive to Ca^2+^: binding to RHBDL2 of WT and G98S Orai1 was reproducibly sensitive to short-term chelation of extracellular Ca^2+^ (Figure 7D).

We next probed the nature of the interaction between Orai1 and RHBDL2. We found that RHBDL2 binding and recognition of Orai1 depended primarily on transmembrane regions and did not require cytoplasmic regions in RHBDL2, nor did it require the C-terminal Stim1-interacting domain of Orai1 (Figures S6E–S6G). Although not dependent on these regions, we found that binding to Orai1 and Orai1ΔCT was strongly enhanced by deletion of the initial 31 amino acids of the RHBDL2 cytoplasmic domain (Figures S6F and S6G). This highlights a crucial region in the amino terminus of RHBDL2 that limits RHBDL2 recognition of Orai1, which we presume is overcome when Orai1 becomes activated. Combining the Ca^2+^ sensitivity results with these domain requirements leads us to conclude that cytoplasmic Ca^2+^ likely contributes to RHBDL2 recognition of Orai1 and, crucially, that the TMDs of both proteins predominantly drive their interaction.

The TMDs of Orai1 undergo large rearrangements upon activation, so we asked whether RHBDL2 uses these conformational changes in the membrane domain to distinguish the active form of Orai1. The structure of the constitutively active *Drosophila* Orai H206A mutant (H134A in human Orai1) is believed to mimic the conformation of Orai1 in complex with Stim1, showing a major displacement of TMD4 (Hou et al., 2018) (Figure 7D). This activating displacement pivots on a flexible hinge generated by a proline residue in TMD4 (proline-245 in human Orai1) (Hou et al., 2012, 2018). Mutation of this hinge proline causes human Stormorken syndrome, in which the CRAC channel has excess activity (Nesin et al., 2014). As helical instability, often conferred by prolines, is a major determinant of rhomboid substrates (Urban and Freeman, 2003; Moin and Urban, 2012), we examined the role of proline-245 in RHBDL2 recognition of Orai1. Strikingly, we found that enhanced rhomboid binding to the active H134A mutant was lost when it was combined with the proline-245-to-leucine mutation (P245L; (Endo et al., 2015) (Figure 7F). Furthermore, we found that the cleavage of both endogenous and overexpressed activated Orai1 was lost upon mutation of P245L (Figure 7G). Of relevance, unlike all other active Orai1 mutants we tested, the P245L mutant did not show enhanced binding to RHBDL2-SA, indicating that even when the molecule is in a locked-open state, proline-245 is necessary for RHBDL2 recognition (Figure S7A). Reinforcing the idea that RHBDL2 recognizes a combination of the active Orai1 conformation and Ca^2+^ influx, the P245L mutation also prevented recognition of the activating G98S mutation (Figures S7A and S7B). We speculate that the G98S mutation in TMD1, which opens the CRAC channel pore in an unregulated manner, may also lead to knock-on changes in the Orai1 structure, which RHBDL2 senses via TMD4. Overall, these data demonstrate that RHBDL2 recognizes active CRAC channels via a helix-breaking proline residue in TMD4.

Finally, RHBDL2 cleavage of the isolated Orai1 TMD4 was inhibited by the P245L mutation (Figure 7H), further confirming the determining role proline-245 plays in RHBDL2 recognition and cleavage of Orai1. In the context of full-length Orai1, the P245L mutation alone led to aberrant proteolysis by exogenous RHBDL2: the cleavage site appeared to shift out of the TMD region, toward the TMD3-4 loop in Orai1 (Figure S7C). Significantly, adding this TMD3-4 loop region to the AP-TMD4 reporter partially restored cleavage by RHBDL2, confirming that ectopic cleavage can occur with this disease mutant (Figure S7D). In line with this, a form of Orai1 with a mutated cleavage site (TMD-F9, as in Figure S1E) led to an even larger shift in the cleavage site (Figure S7C). Whether these changes in exogenous RHBDL2 cleavage site preference are physiologically important warrants further attention: a shift in the cleavage site may have an impact on the normal effect of proteolysis. Moreover, these data extend a growing theme that once rhomboids recognize substrate, they irreversibly commit to their cleavage (Urban and Freeman, 2003; Akiyama and Maegawa, 2007; Strisovsky et al., 2009; Moin and Urban, 2012; Baker and Urban, 2015).

Overall, these data demonstrate that loss of helical instability in TMD4, which activates Orai1 and causes Stormorken syndrome, also blocks recognition by rhomboid and thus has a negative impact on its ability to perform conformational surveillance.

## Discussion

The results we report here uncover an unexpected role for rhomboid proteases in regulated degradation of membrane proteins. RHBDL2 patrols the PM to seek and destroy inappropriately activated CRAC channels in unstimulated cells, in order to prevent inappropriate signaling, exemplified by the severely dysregulated NFAT-dependent expression of the proinflammatory cytokine, TNF. RHBDL2 thus maintains a low baseline level of Ca^2+^ influx, which is essential to ensure fully regulated switch-like SOCE, for example in T cell activation. It is notable that RHBDL2 has been shown to diffuse in the plane of the membrane at exceptionally high speeds, faster than any known polytopic membrane protein (Kreutzberger et al., 2019). This property, which appears to be mediated by mismatch between the thickness of the lipid bilayer and the shorter than expected hydrophobic domain of the rhomboid fold, makes RHBDL2 particularly well suited to this surveillance function. We discovered Orai1 using a combined bioinformatic and cell-based screen for substrates of the PM rhomboid RHBDL2. This approach identified specifically the fourth TMD of Orai1 (as well as Orai2 and Orai3). Combined with the recent discovery of a polytopic substrate for the bacterial rhomboid protease YqgP (Began et al., 2020), the universe of possible rhomboid substrates has therefore been expanded beyond single-pass TMD proteins, to now encompass the very large class of polytopic membrane proteins, which includes channels, GPCRs, and many other pharmacologically significant targets.

Prolines are often determinants of rhomboid substrates, because of their property of destabilizing or introducing a kink into a transmembrane helix (Urban and Freeman, 2003). This partial disruption is needed to allow the active site of the enzyme access to the cleavable peptide bond, which is otherwise shielded by hydrogen bonding inherent to the alpha helix. Our data elaborate on this basic principle of rhomboid substrate recognition by identifying a case in which a rhomboid shows conformational specificity in substrate recognition. We are unaware of other examples of this among intramembrane proteases. RHBDL2 monitors the conformational dynamics of Orai1. Proline-245 of Orai1 contributes the flexibility to the fourth TMD that is essential for transducing the binding of Stim1 into the allosteric changes that open the CRAC channel. This same proline-245 hinge mechanism determines recognition by RHBDL2. The ability to distinguish active from inactive Orai1 underlies the ability of RHBDL2 to cleave activated CRAC channels, and is therefore central to the mechanism of maintaining low basal signaling in unstimulated cells. Interestingly, transmembrane helix instability is a common characteristic of most intramembrane protease substrates (Ye et al., 2000; Lemberg and Martoglio, 2002; Urban and Freeman, 2003), which raises the possibility that other intramembrane proteases may perform a similar function.

Overall, we propose that the cycle of spontaneous CRAC channel activation/inactivation in cells at rest is suppressed by RHBDL2 activity. Upon spontaneous activation of a CRAC channel, RHBDL2 is recruited by the conformational change in Orai1 TMD4 and perhaps also the enhanced local Ca^2+^ flux across the membrane. Upon RHBDL2 cleavage of Orai1, TMD4 is destroyed and Ca^2+^ influx is blocked, allowing RHBDL2 to dissociate. This substrate selectivity leaves the vast majority of Orai1 at the cell surface untouched, ready for normal activation by SOCE. This model that RHBDL2 acts to prevent inappropriate CRAC channel activity begs the question of how stimulated activity occurs when SOCE appropriately triggers signaling. What prevents RHBDL2 from blocking signaling when signaling is needed? We propose two possible answers to explain this. First, SOCE leads to molecular crowding of CRAC channels in the membrane, with an estimated ∼40 nm distance between channels (Ji et al., 2008). Such a high density of CRAC channels may physically restrict RHBDL2 access to substrate TMDs within the plane of the membrane. Second, in unstimulated cells only a small proportion of Orai1 encounters Stim1, allowing the low level of RHBDL2 expressed in most cells to be sufficient to prevent unstimulated signaling (our observations and https://gtexportal.org/home/). In contrast, when SOCE is triggered, the majority of Orai1 is engaged, and this may simply overwhelm RHBDL2 surveillance.

CRAC channel activity is a major mechanism for regulating cytoplasmic Ca^2+^ levels in non-excitable cells and therefore plays an important role in a wide range of biological contexts, most notably during the activation of T cells when they engage with antigen-presenting cells (Feske, 2007). Although SOCE, dependent on phospholipase C activity, is a tightly regulated process, even highly evolved biological control processes are not perfect. The events downstream of CRAC channels are biologically potent, and if unchecked, they cause pathophysiological dysregulation and channelopathies (Feske, 2010). Our results with Orai1 tagged with a Ca^2+^ influx sensor show that there is indeed a detectable level of channel opening in resting cells, and that RHBDL2 acts to dampen this inappropriate signaling: its loss leads to significantly elevated levels of Orai1, and consequent dysregulated T cell activation and inflammatory cytokine expression. Significantly, the major determinant of RHBDL2 recognition of Orai1, proline-245, is the causative mutation of the rare inherited Stormorken syndrome, which is characterized by excess CRAC channel activity (Nesin et al., 2014). The etiology of this disease, and perhaps others caused by excess CRAC channel activity, is therefore likely to be related to failure of RHBDL2 surveillance of Orai1.

In conclusion, the identification of Orai1 as a substrate of RHBDL2 highlights two themes. First, it substantially advances our knowledge of rhomboid proteases by expanding the universe of potential substrates and, by demonstrating intramembrane proteases show conformation-specific substrate recognition, has significant implications for their regulatory roles. Second, our work also develops a theme of regulated protein degradation being used to sharpen cellular signaling, by ensuring low levels of activity in unstimulated cells. RHBDL2 patrols the PM, seeking Orai1 molecules in an inappropriately active conformation in resting cells and triggering their degradation. Rhomboid proteases are ancient, existing in all kingdoms of life. It is tempting to speculate that the primordial function of rhomboids may have been to inactivate and degrade membrane proteins with non-canonical TMDs, perhaps as a quality control function. In this scenario, it would only have been later, after the appearance of metazoans, when rhomboids would become responsible for their now well established roles in triggering the release of proteins that signal between cells.

### Limitations of the study

The proposed model is based predominantly on western blot and light microscopy data in model cell lines expressing recombinant versions of Orai1 and RHBDL2. In most cases we have aimed to confirm our findings with endogenous proteins at physiological levels and through loss-of-function experiments in multiple relevant cell lines and primary T cells. However, additional work is required to extend the physiological relevance of our model, such as development of animal models specific to our findings. Furthermore, our work reveals that RHBDL2 has strongly diminished ability to recognize and cleave a Stormorken syndrome-causing mutant Orai1 (P245L). Because of the rarity of this disease, we were unable to test whether Orai1 cleavage is inhibited in patient cells. Last, our screen for rhomboid protease substrates highlights many potential substrates, but only a handful of these have been fully validated, so further work is required to confirm these as true substrates.

## STAR★Methods

### Key resources table

**Table:** 

REAGENT or RESOURCE	SOURCE	IDENTIFIER
**Antibodies**		

Mouse anti-HA tag (16B12)	ENZO	ENZ-ABS120-0200
Mouse anti-beta-actin	Santa Cruz	sc-47778; RRID:AB_626632
Mouse anti-Transferrin Receptor (clone H68.4)	Invitrogen	13–6800; RRID:AB_86623
Rabbit anti-Stim1 (D88E10)	Cell Signaling Technology	5668S
Rabbit anti-Orai1	Sigma Aldrich	O8264; RRID:AB_1078883
Goat anti-Myc tag	Abcam	ab9132; RRID:AB_307033
Rabbit anti-RHBDL2	Proteintech	12467-1-AP; RRID:AB_11232403
Rabbit anti-V5 tag (D3H8Q)	Cell Signaling Technology	13202S; RRID:AB_2687461
Anti-goat-HRP, mouse monoclonal	Santa Cruz	sc-2354; RRID:AB_628490
Anti-mouse-HRP, goat polyclonal	Santa Cruz	sc-2055; RRID:AB_631738
Anti-rabbit-HRP, goat polyclonal	Sigma-Alrich	A9169; RRID:AB_258434

**Bacterial and virus strains**		

Stellar Competent Cells	Takara	636766
One-shot Stbl3 Chemically Competent E.coli	Invitrogen	C737303

**Chemicals, peptides, and recombinant proteins**		

Bafilomycin A1	Sigma Aldrich	19-148
Biotin	Sigma Aldrich	B4639
Blasticidin	Sigma Aldrich	15205
Thapsigargin	Sigma Aldrich	T9033
Ionomycin Calcium Salt	Peprotech EC Limited	5608212-1mG
Doxycycline	MP Biomedicals	SKU 0219504405
PNGase F	New England Biolabs	P0704L
Puromycin	GIBCO	A11138-03
Zeocin	Invitrogen	2058442
CD3	eBioscience	UCHT1
anti-CD69-allophycocyanin (APC)	Life Technologies	MHCD6905

**Critical commercial assays**		

Rosette Sep	Stem Cell Technologies	15705
Dynabeads Mouse T-Activator CD3/CD28	Thermo Fisher	11456D
TaqMan Gene Expression Assay System		N/A
Phosphatase substrate kits containing PNPP tablets	Thermo Scientific	37620

**Experimental models: Cell lines**		

HEK293T cells	This paper	RRID:CVCL_0063
R2 KO HEK293T cells	Freeman lab; this paper	N/A
HaCaT cells	This paper	RRID:CVCL_0038
R2 KO HaCaTs	Stříšovský lab; this paper	Clone H9 (Control clone B10)
Inducible V5-Orai1/Stim1-BirA OE HaCaT cells	Freeman lab; this paper	N/A
Primary CD4 positive T cells	National Health Service Blood and Transplant (NHSBT)	N/A
Inducible V5-Orai1/Stim1-BirA OE R2 KO HaCaT cells	Freeman lab; this paper	N/A

**Oligonucleotides**		

Human Orai1 siRNA	Horizon	L-014998
Human RHBDL1 siRNA	Invitrogen	HSS113329; HSS113330
Human RHBDL2 siRNA	Invitrogen	HSS123556; HSS123558
Human RHBDL3 siRNA	Invitrogen	HSS136312; HSS136314
Human RHBDL4 siRNA	Invitrogen	HSS130774; HSS130775
Human Stim2 siRNA	Invitrogen	HSS183972; HSS183973
Negative control siRNA	Invitrogen	462001
Applied Biosystems TaqMan RHBDL2 probe	Life Technologies	Hs00983274_m1
Applied Biosystems TaqMan Stim2 probe	Life Technologies	Hs00957788_m1
Applied Biosystems TaqMan TNF probe	Life Technologies	Hs00174128_m1
Applied Biosystems TaqMan IL-6 probe	Life Technologies	Hs00985639_m1
Applied Biosystems TaqMan GAPDH probe	Life Technologies	Hs02786624_g1

**Recombinant DNA**		

pLKO.1 RHBDL2 shRNA Lentiviral Vectors	Adrain et al., 2011	CA143, CA146, CA148, CA149
pCMV delta8.91	Adrain et al., 2011	N/A
pMD.VSVG	Adrain et al., 2011	N/A
pcDNA3.1_TMDscreen (with TMDs in Table S1)	This paper	N/A
GFP-NFAT1	Kar et al., 2011.	N/A
Orai1-myc	Yeh et al., 2020	N/A
Stim1-YFP	Yeh et al., 2020	N/A
pcDNA3.1 V5-hOrai1	This paper	WT, G98C, G98S, R91W, F99Y, V102A, H134A, H134T, H134V, H134S, P245L, G98S/P245L, H134A/P245L, TMD-F9, ΔCT
pLVX V5-hOrai1	This paper	N/A
pLVX Stim1-BirA	This paper	N/A
pCDNA3.1 RHBDL1-4	Lohi et al., 2004	WT and SA mutants
pCDNA3.1 RHBDL2 deletion mutant	This paper	ΔN short and ΔN internal
G-GECO	Yu-Fen Chang; this paper	N/A
Orai1-K-GECO	Yu-Fen Chang; this paper	N/A
pLenticrisprv2 with guides for RHBDL2:	Sanjana et al., 2014	pPR62, pPR63
CCAAGAGTAAAAAGGTCCAC		
or		
ATGCTGCCCGAAAAGTCCCG		
px462-SpCas9n targeting regions in RHBDL2:	This paper; (Ran et al., 2013)	Addgene plasmid #62987
TGAGCTGCAAAAGACACCTTGGG(-)		
or		
GGATTTGCTGGAATGTCCATTGG(+)		

**Software and algorithms**		

ImageJ	(Schneider et al., 2012)	https://imagej.nih.gov/ij/
HHpred	Zimmermann et al., 2018	https://toolkit.tuebingen.mpg.de/tools/hhpred
Uniprot	The Uniprot consortium; Nucleic Acids Res. 49:D1 (2021)	https://www.uniprot.org),
Clustal Omega	EMBL-EBI	https://www.ebi.ac.uk/Tools/msa/clustalo/
FlowJo (version X 10.0.7r2)	FlowJo, LLC	https://www.flowjo.com/solutions/flowjo
Prism (version 7)	GraphPad	https://www.graphpad.com/scientific-software/prism/
NIS-Element system	Nikon, Japan	http://www.microscope.healthcare.nikon.com/products/software/nis-elements
TMHMM	(Krogh et al., 2001)	http://www.cbs.dtu.dk/services/TMHMM/)

### Resource availability

#### Lead contact

Further information and requests for reagents should be directed to and will be fulfilled by the Lead Contact, Matthew Freeman (matthew.freeman@path.ox.ac.uk).

#### Materials availability

All unique reagents generated in this study are available from the Lead Contact without restriction.

### Experimental model and subject details

#### Cell culture

All cell lines in this study were maintained in regular high-glucose DMEM, supplemented with 10% FCS, 100 μg/ml penicillin, and 100 μg/ml streptomycin. All cells used in this study were subject to regular mycoplasma testing.

#### T cell isolation

T cells were isolated as previously described (Breuning and Brown, 2017). In brief, using RosetteSep, primary CD4^+^ T cells were isolated from cones from anonymous human blood donors with approval from the National Health Service Blood and Transplant (NHSBT) and stimulated with CD3 and CD28 mAbs on Dynabeads (Thermo Fisher) in the presence of 100 U/ml IL-2 to produce T cell blasts. Primary T cell blasts were transduced with pLKO-based shRNA expressing lentivirus and selected for puromycin resistance for at least one week. Depletion of RHBDL2 mRNA was then confirmed by RT-pPCR.

### Method details

#### Reagents

Bafilomycin A1 (catalog number 19-148), biotin (catalog number B4639), blasticidin (catalog number 15205) and thapsigargin (catalog number T9033) was purchased from Sigma Aldrich. Doxycycline was purchased from MP Biomedicals (catalog number SKU 0219504405). PNGase F was purchased from New England Biolabs (catalog number P0704L). Puromycin was purchased from GIBCO (catalog number A11138-03). Zeocin was purchased from Invitrogen (catalog number 2058442). Phosphatase substrate kits containing PNPP tablets and buffer (catalog number 37620) were purchased from Thermo Scientific.

#### Antibodies

The following antibodies were used for western blotting (WB) and immunofluorescence (IF): mouse anti-beta-actin (Santa Cruz, catalog number sc-47778; WB 1:2000), mouse anti-HA (ENZO, catalog number ENZ-ABS120-0200; WB 1:1000), mouse anti-transferrin receptor (Invitrogen, catalog number 13–6800; WB 1:1000), rabbit anti-Stim1 (Cell Signaling Technology, catalog number 5668S (D88E10); WB 1:2000), rabbit anti-Orai1 (Sigma Aldrich, catalog number O8264; WB 1:2500), goat anti-myc tag (Abcam, catalog number ab9132; IF 1:2000), rabbit anti-RHBDL2 (Proteintech, catalog number 12467-1-AP; WB 1:250 – only detected RHBDL2 in HaCaT lysates), rabbit anti-V5 tag (Cell Signaling Technology, catalog number 13202S; WB and IF 1:2000). Corresponding species-specific HRP or fluorescently coupled secondary antibodies were used from Santa Cruz and Cell Signaling (WB) or Invitrogen (IF).

#### DNA plasmids

For generation of the AP reporter construct, we PCR amplified sequence encoding the signal sequence of HB-EGF and alkaline phosphatase, as described originally in (Sahin et al., 2004), with a pair of restriction enzyme sites (SalI and NotI) that were placed 3′ of the sequence encoding alkaline phosphatase. This was subsequently cloned between the EcoRI and SalI sites in the multiple cloning site of pcDNA3.1. This created a construct (pcDNA3.1_TMDscreen) that expresses a protein that constitutively enters the endoplasmic reticulum. Sequences encoding candidate TMDs, plus 3 cytoplasmic amino acids and 7 extracellular amino acids, were then ordered as paired oligonucleotides with 20 base pairs of overlap, and with overhangs that complemented the SalI and NotI sites in pcDNA3.1_TMDscreen. Paired oligonucleotides were extended on one another with 3 rounds of 98°C (15 s) → 55°C (20 s) → 72°C (30sec), followed by 72°C (7 min) in a thermal cycler. Double stranded oligos were then column purified and cloned into SalI-NotI digested pcDNA3.1_TMDscreen by InFusion cloning, test digested and positive colonies were confirmed by Sanger sequencing (Source Bioscience). pcDNA3.1 vectors encoding 3xHA-mRHBDL1-4 and GFP-mRHBDL2 have been previously described elsewhere (Lohi et al., 2004; Adrain et al., 2011). Inactive S- > A mutants, and AP-TMD4 Orai1 point mutant were generated using site directed mutagenesis kits, according to the manufacturers instructions (Agilent). Stim1-YFP and GFP-NFAT1 (1-460) were previously used and described in Kar et al., 2011. Orai1-myc was previously described in Yeh et al., 2020. To generate stable HaCaT cell lines inducibly expressing V5-Orai1 and Stim1-BirA we used pLVX plasmids (Takara) that were subcloned to express zeocin and blasticidin resistance genes, a kind gift from Dr Michael van der Weijer (Dunn School, Oxford). To generate pcDNA3.1-V5-Orai1 and pLVX-V5-Orai1, we PCR amplified human Orai1 either flanked by the regions surrounding the BamHI/XhoI sites in pcDNA3.1 or the AgeI site in pLVX_Blasticidin. After digestion of pcDNA3.1 and pLVX_Blasticidin with corresponding restriction enzymes, we then inserted V5-Orai1 by InFusion cloning, according to manufacturers instructions (Takara). To generate pLVX-Stim1-BirA, we PCR amplified human Stim1 from Stim1-YFP, and BirA^∗^ (Roux et al., 2012), with 20 nucleotides of overlap with one another and the sequence flanking the AgeI site in pLVX_Zeocin. After digestion of pLVX_Zeocin with AgeI, we then inserted Stim1-BirA^∗^ by InFusion cloning, according to the manufacturers instructions (Takara).

#### CRISPR-Cas9 gene editing

For all editing, sequences of suitable guide RNAs were designed using publicly available prediction tools at https://www.broadinstitute.org/rnai/public/analysis-tools/sgrna-design (Doench et al., 2014) and at https://zlab.bio/guide-design-resources (Hsu et al., 2013). A paired nickase Cas9 strategy was used to target the catalytic histidine in HEK cells to generate RHBDL2 KO HEKs (Figures S2A and S2B). Guides targetting the following loci in human chromosome 1: TGAGCTGCAAAAGACACCTTGGG(-) and GGATTTGCTGGAATGTCCATTGG(+) were chosen. Guide sequences, without the PAM, were cloned into pSpCas9n(BB)-2A-Puro (px462), and sequence verified. Cells grown in 6 well dishes at 30%–40% confluency were transfected with 500 ng CRISPR/Cas9. 24 h later cells were selected in 1 μg/ml puromycin (GIBCO) overnight. After 24 h recovery, 100 cells were seeded for colony growth in 10cm dishes. Colonies were picked used cloning discs and cells were amplified in 24 well dishes. Expanded colonies were then lysed at 65°C in 10 mM Tris-HCl (pH 8), 25 mM NaCl, 1mM EDTA, and 200 μg/ml proteinase K. Proteinase K was inactivated at 95°C for 2 min and samples were analyzed via PCR and high resolution melt analysis, according to (Bassett et al., 2013), and deletions were confirmed by Sanger sequencing (Source Bioscience, Oxford). Clone “5j” was found to contain a deletion around the catalytic histidine, which would also produce a premature stop codon. To target the endogenous RHBDL2 gene in HaCaT cells using CRISPR/Cas9 we introduced a premature stop codon within exon 2 of the endogenous RHBDL2 gene by introducing indels targeting a site 115 bp after the initiator ATG codon of RHBDL2 (Figures S2C and S2D). Two of the four highest scoring guide RNA sequences (gRNA4: CCAAGAGTAAAAAGGTCCAC and gRNA1: ATGCTGCCCGAAAAGTCCCG) were cloned into pLenticrisprv2 (Sanjana et al., 2014) to yield targeting constructs pPR62 and pPR63, respectively. HaCaT cells were seeded at 5 × 10^5^ cells per 6 cm dish. The next day cells were transfected with 2 μg pPR62 or pPR63 using Fugene 6. 24 h later cells were selected using 2 μg/ml puromycin for 96 hr. Then media was exchanged for complete DMEM and cells were allowed to recover to confluence, at which point they were trypsinized, suspended in 1 mL PBS + 2% FBS and single cell sorted into 96 well plates containing 20% FBS, 50% conditioned medium, 30% complete medium and 1 × Gentamycin/AmphotericinB (1 μg/ml and 250 ng/ml, respectively; Thermo R01510). Single cells were allowed to proliferate and were expanded for analysis by indel screening and western blotting. To screen for indels, genomic DNA from each clone was isolated, amplified by PCR using primers flanking the Cas9 cleavage site, and products were analyzed by TBE agarose electrophoresis and comparison to untargeted HaCaT cells. Positive clones containing indels and lacking the wild-type allele were further characterized by DNA sequencing and western blotting. For sequencing, primers flanking the Cas9 cleavage site were used to amplify this region by PCR from the genomic DNA of candidate KO clones before cloning into a vector, transformation and propagation in *E.coli*. A minimum of 7 colonies per clone were sequenced to identify all possible genomic alterations at the Cas9 cleavage site in the hypotetraploid HaCaT cells. Ultimately, absence of endogenous RHBDL2 protein and enzymatic activity was verified by western blotting using a polyclonal antibody against human RHBDL2 and an RHBDL2 substrate (Figure S2D). To serve as controls, untargeted wild-type HaCaT cells were single-cell sorted and expanded into clonal cultures. Wild-type clone B10 and RHBDL2 deficient clone H9 (generated with gRNA4) were used in all experiments in Figures 6C–6F.

#### Lentivirus production and transduction

HEK293 cells grown to 30%–40% confluence in 6-well dishes were transfected with Lipofectamine 2000 (Invitrogen) in 35mm plates with 0.5 μg of pLKO shRNA or pLVX expression plasmids (Takara), 0.35 μg pCMV-dR8.2 and 0.15 μg pCMV-VSVG. The pLKO shRNA plasmids (Control shRNA against RhoGDI: CA143; RHBDL2 #1: CA146; RHBDL2 #2: CA148; RHBDL2 #3: CA149) were previously validated by (Adrain et al., 2011). The following day, medium was changed and transfected cells were allowed to secrete virus for 48-72 hours in 2 mL complete medium. Culture supernatants were then centrifuged clarified by filtration with Sartorius Minisart syringe filters (0.45 μm pore size). For infection of HaCaTs or primary CD4-positive T cells, cells were split the day before, and viral supernatants were diluted 1- or 2-fold in fresh medium for transduction. Transduction was carried out in the presence of 10 μg/ml polybrene and a medium change was made 24 hours later. For selection, cells were treated with 10 μg/ml puromycin, 100 μg/ml zeocin or 5 μg/ml blasticidin, until all cells were killed in control transductions. In the case of the Stim1-BirA^∗^ HaCaTs, WT and KO cells were first transduced with pLVX-V5-Orai1-myc (and selected with blasticidin), followed by a second transduction with pLVX-Stim1-BirA^∗^ (and selected with zeocin).

#### siRNA

Orai1 siRNA was purchased from Horizon, ON-TARGET SMARTPool (Catalog ID: L-014998). A final concentration of 50 nM was used to knockdown Orai1. The following siRNAs against human rhomboid proteases were purchased from Invitrogen: RHBDL1 #1: HSS113329; RHBDL1 #2: HSS113330; RHBDL2 #1: HSS123556; RHBDL2 #2: HSS123558; RHBDL3 #1: HSS136312; RHBDL3 #2: HSS136314; RHBDL4/RHBDD1 #1: HSS130774; RHBDL4/RHBDD1 #2: HSS130775; Stim2 #1: HSS183972; Stim2 #2: HSS183973. Negative control medium GC duplex (cat no: 462001) was purchased from Invitrogen. For rhomboid knockdowns, including where two siRNAs were combined, a final concentration of 75 nM was used. siRNAs were delivered using Lipofectamine RNAiMAX, according to the manufacturer’s instructions (Invitrogen), and incubated for indicated time periods.

#### AP-TMD shedding assay

To test rhomboid cleavage of candidate substrate transmembrane domains, 5 × 10^4^ HEK293 cells were plated in one well of a 96 well plate in the presence of 30 ng each of plasmids encoding AP-TMD and 3xHA-RHBDL constructs, pre-complexed in optiMEM (GIBCO) with FuGene6 HD transfection reagent (Promega), according to manufacturers instructions. Cells were left for 24 hours to attach and express protein, and then exchanged into 200 μl optiMEM overnight. AP activity was detected in the supernatants or in cell lysates (using Triton X-100 buffer) by adding equal volumes of PNPP buffer (Thermo Scientific) followed by measurement of absorbance at 405 nm on a plate reader. The percentage of the total material shed from each well (i.e., signal from supernatant divided by total signal from lysate and supernatant) was then used to calculate release, and processed as described in the figure legends. Error bars represent SEM.

#### Cytosolic calcium readouts (including barium)

Cells were loaded with Fura 2 by incubating in 1 μM Fura 2-AM in external solution (145 mM NaCl, 2.8 mM KCl, 2 mM CaCl2, 2 mM MgCl2, 10 mM Dglucose, 10 mM HEPES, pH 7.4) for 40 minutes in the dark, followed by washing and incubating in external solution for another 15 minutes for full de-esterification. Ca^2+^-free solution comprised of 145 mM NaCl, 2.8 mM KCl, 2 mM MgCl2, 10 mM D-glucose, 10 mM HEPES, 0.1 mM EGTA, was applied to cells prior to Ca^2+^ image measurement, 2 μM of thapsigargin diluted to a final volume of 10 μl by Ca^2+^-free solution was applied in around 1 min after the recording started. While the trace go to the basal levels in around 10 min after Thapsigargin treatment, 2mM Ca^2+^ or Ba^2+^ were then applied. Cells were alternately excited at 356 and 380 nm, and signals were acquired every 2 s. Calcium signals are represented by the 356 nm/380 nm ratio (R). All the images were analyzed by using IGOR Pro software. All Fura-2 imaging data was calibrated for Ca^2+^ concentration with the NIS-Element system (Nikon, Japan), based on (Grynkiewicz et al., 1985; Kong and Lee, 1995).

#### Spontaneous CRAC channel activity assay

HaCaT cells treated with control or RHBDL2 siRNA were transfected with Orai1-K-GECO and G-GECO using Lipofectamine 3000 and subsequently imaged on an inverted microscope (DMi8, Leica) equipped with a 63x objective lens (N.A. 1.4) and a multiwavelength LED light source (pE-4000, CoolLED) in regular Ca^2+^ containing solution. Media replacement was followed by imaging with an iXon EMCCD camera (Andor) using 488 nm LED illumination. The GFP filter set (DS/FF02-485/20-25, T495lpxr dichroic mirror, and ET525/50 emission filter) was used for G-GECO observation. The RFP filter set (excitation 545/30, 565 nm long-pass dichroic mirror, emission 620/60 nm) was used to visualize K-GECO. Time-lapse images were taken at either 0.3 or 0.5 Hz for both Orai1-K-GECO and G-GECO. Where indicated cells were either incubated in Ca^2+^-free solution containing 0.1 mM EGTA for 20 mins prior to imaging, or pre-treated with 10 μM BTP2 for 30 mins prior to imaging to block CRAC channel activity. An average baseline value of Orai1-K-GECO fluorescence intensity over time was calculated, and in subsequent analysis a > 20% increase in that baseline over time was defined as spontaneous CRAC channel activation, as this was the value that consistently correlated with an increase in cytosolic Ca^2+^, as reported by G-GECO.

#### T cell activation assay

96-well round-bottom plates were coated with varying doses of CD3 mAb (UCHT1; eBioscience). Transduced activated primary CD4^+^ T cells (5 × 10^5^ cells in 200 μl) were added, and the mixture was incubated for 18 h at 37°C. Cells were then stained with anti-CD69-allophycocyanin (APC) (Life Technologies) and analyzed for percentage positivity by flow cytometry using a FACSCalibur plate reader (BDBiosciences). Dose-response curves and EC_50_ values were generated with GraphPad Prism.

#### qRT-PCR

Total RNA was isolated from HaCaT cells by using RNeasy micro kit (QIAGEN) according to the manufacturers instructions. 2000 ng RNA was used for cDNA synthesis using a cDNA Synthesis kit (PCR Biosystems) according to the manufacturers instructions. In most cases, the cDNA was then diluted 5-fold in water, except from cDNA from primary T cells, which was undiluted. qPCR was performed using TaqMan gene expression assays (Applied Biosystems) against the stated target genes in a StepOnePlus system (Applied Biosystems). GAPDH was used as a housekeeping gene for normalization. The Applied Biosystems TaqMan probes, all purchased through Life Technologies, were as follows: RHBDL2 (Hs00983274_m1), TNF alpha (Hs00174128_m1), IL-6 (Hs00985639_m1), Stim2 (Hs00957788_m1) and GAPDH (Hs02786624_g1).

#### SDS-PAGE and western blotting

Samples were typically electrophoresed at 150V on 4%–12% Bis-Tris gels (Invitrogen) until the dye front had migrated off the gel (approx. 10-15 kDa). Gels were transferred onto PVDF membranes and blocked in PBS or TBS containing Tween 20 (0.05%) and 5% milk or 1% BSA, before detection with the indicated primary antibodies and species-specific HRP-coupled secondary antibodies. Band visualization was achieved with Enhanced Chemiluminescence (Amersham Biosciences) using X-ray film. To aid quantification of Orai1 protein, all Orai1 lysate preparations were treated with PNGase (NEB) to remove all glycosylation, according to the manufacturers instruction.

#### Stim1-BirA^∗^ biotin capture assay

WT and R2 KO HaCaT cells expressing pLVX-based V5-Orai1-myc and Stim1-BirA^∗^ were plated at 1 × 10^6^ in the presence of 50 μM biotin and 100 ng/ml doxycycline (to induce their expression) for 96 hours. Prior to lysis, where stated, cells were then treated with 100 nM bafilomycin A1. Cells then underwent 3x PBS washes to remove excess biotin. Cells were then lysed in RIPA buffer (50mM Tris pH 7.4, 150 mM NaCl, 1% NP40, 0.5% Sodium Deoxycholate, pH 7.4) containing complete protease inhibitor cocktail (Roche). Lysates were pulse-sonicated in an ice-water bath for 5 mins. After pelleting at 10,000 x g, clarified supernatants were incubated with 30 μL high-capacity neutravidin agarose beads overnight to capture biotinylated proteins (Thermo Scientific, catalog number 29204). Beads were then washed 3x with ice-cold RIPA buffer and eluted with 2x SDS sample buffer with excess biotin at 95°C for 15 mins. In all cases, 50% of the bead eluate and 1% lysate was loaded onto SDS-PAGE gels.

#### Immunoprecipitation

HEK293 cells transfected for 24 hours with different versions of V5-Orai1-myc and 3xHA-RHBDL2-SA were grown to ∼90% confluence in 10 cm plates, on the day of IP. Cells were washed 3x with PBS and then lysed in 1 mL TX-100 lysis buffer (1% Triton X-100, 150mM NaCl, 50 mM Tris-HCl, pH 7.4) supplemented with protease inhibitor cocktail (Roche). Cell lysates were cleared by centrifugation at 10,000 x g for 10 mins at 4°C. Protein concentrations were measured by a BCA assay kit (Pierce). The lysates were then immunoprecipitated for 2-3 hours with 20 μL pre-washed HA antibody-coupled beads at 4°C on a rotor. After 4-5 washes with lysis buffer, the immunocomplexes were incubated at 65°C for 15 mins in 2x SDS sample buffer. Typically, 50% of the immunoprecipitates and 1% of lysates were resolved on SDS-PAGE gels for subsequent western blotting.

#### Light microscopy

HEK293 cells transfected with indicated constructs were plated on 13mm glass coverslips in 6 well dishes. Cells were washed 1x in room temperature PBS and fixed with 4% paraformaldehyde in PBS at room temperature for 20-30 mins. Fixative was quenched with 50mM NH_4_Cl for 5 mins. Cells were permeabilised in 0.2% TX-100 in PBS for 30 mins and epitopes blocked with 1% fish-skin gelatin (Sigma) in PBS for 1 hour. Coverslips were then incubated overnight with indicated antibodies in 1% fish-skin gelatin/PBS. After 3x PBS washes, coverslips were incubated with corresponding species-specific fluorescently coupled secondary antibodies (Invitrogen) for 45mins. Cells were subsequently washed 3x with PBS and 1x with H_2_O, prior to mounting on glass slides with mounting medium containing DAPI (ProLong Gold; ThermoFisher Scientific). For GFP-NFAT experiments, the fluorescent GFP signal was acquired. Images were acquired with a laser scanning confocal microscope (Fluoview FV1000; Olympus) with a 60x1.4 NA oil objective, and processed using Fiji (ImageJ).

#### Bioinformatics

For searches based on TMD helical instability, we used HHpred in the MPI Bioinformatics Toolkit (https://toolkit.tuebingen.mpg.de/tools/hhpred). We queried the mouse proteome using *Drosophila melanogaster* Spitz, using the sequence surrounding and including the transmembrane domain region (PRPMLEKASIASGAMCALVFMLFVCLAFYLRFE). Most of the top hits that had an aligned transmembrane domain in the .hhr file (available upon request) were picked for the TMD screen with mouse RHBDL2. For searches of EGF domain-containing TMD proteins, we used Uniprot (https://www.uniprot.org), selecting for the presence of both transmembrane helices in a Type-I orientation and presence of an extracellular EGF-like domain. TMD regions of the hits were uniformly determined using manual searches in TMHMM Server v.2.0 (http://www.cbs.dtu.dk/services/TMHMM/) and we included three amino acids on the cytoplasmic side, and seven amino acids on the extracellular/luminal side, according to Uniprot amino acid sequence entries (https://www.uniprot.org). RHBDL2 expression data was taken from the GTEx Portal. The Genotype-Tissue Expression (GTEx) Project was supported by the Common Fund of the Office of the Director of the National Institutes of Health, and by NCI, NHGRI, NHLBI, NIDA, NIMH, and NINDS. The data used for the analyses described in this manuscript were obtained from: the GTEx Portal on 01/07/20.

### Quantification and statistical analysis

Densitometry data for western blots were generated using ImageJ. Representative images of at least three independent experiments are shown. Graphs and heatmaps were plotted using Prism (GraphPad). Error bars represent standard error of the mean, or standard deviation (as indicated in figure legends). Statistical significance of the data was mostly assessed by using Student’s t tests, comparing control and test conditions, and is described in the figure legends. The number of individual cells analyzed in all calcium imaging experiments is stated in the figure legends. The T cell data is representative of two human donors, three individual shRNA treatments and three biological replicates.

## Data Availability

• The raw fluorescence microscopy and western blotting data generated during this study have been deposited at Mendeley Data and are publicly available as of the date of publication. Mendeley Data: http://dx.doi.org/10.17632/zgn7zmpfvb.2. • This paper does not report original code. • Any additional information required to reanalyse the data reported in this paper is available from the Lead Contact upon request.

## Supplemental information

Document S1. Figures S1–S7

Table S1. Transmembrane domain sequences used in the screen, related to Figure 1 Amino acid sequences of the TMDs and surrounding regions from indicated proteins that were used in the AP-TMD screen.

Document S2. Article plus supplemental information
