# Supplementary material for: Conformational surveillance of Orai1 by a rhomboid intramembrane protease prevents inappropriate CRAC channel activation
Source: Mol Cell. 2021 Dec 2;81(23):4784–4798.e7. doi: 10.1016/j.molcel.2021.10.025 (PMC8657799; doi:10.1016/j.molcel.2021.10.025)
Supplement: Document S1. Figures S1–S7 [file mmc1.pdf]

Molecular Cell, Volume *81*

Supplemental information

Conformational surveillance of Orai1 by a rhomboid intramembrane protease prevents inappropriate CRAC channel activation

Adam G. Grieve, Yi-Chun Yeh, Yu-Fen Chang, Hsin-Yi Huang, Lucrezia Zarcone, Johannes Breuning, Nicholas Johnson, Kvido Stříšovský, Marion H. Brown, Anant B. Parekh, and Matthew Freeman

Figure S1

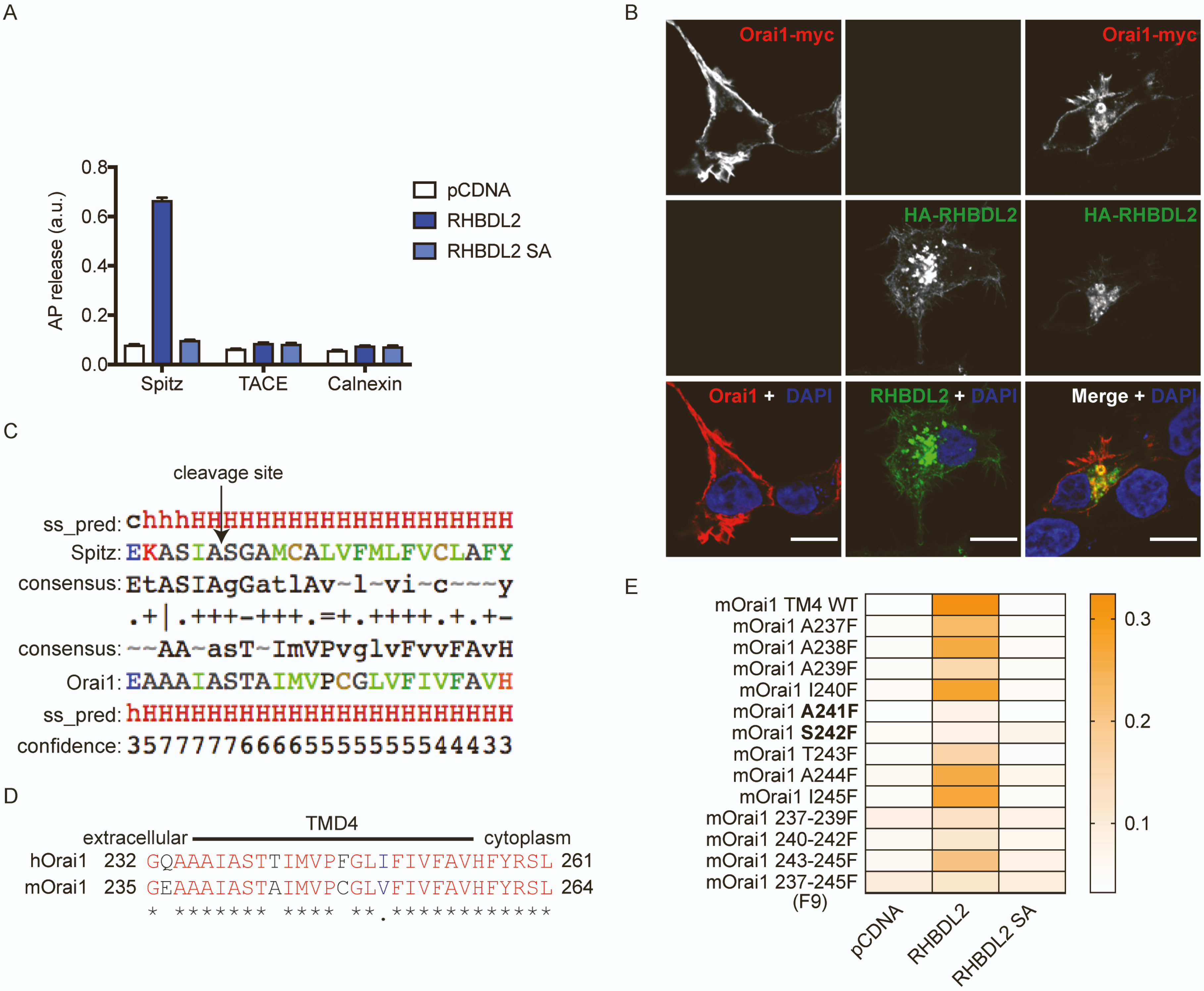
A
AP release (a.u.)
0.0
0.2
0.4
0.6
0.8
Spitz
TACE
Calnexin
pCDNA
RHBDL2
RHBDL2 SA
B
Orai1-myc
Orai1-myc
HA-RHBDL2
HA-RHBDL2
Orai1 + DAPI
RHBDL2 + DAPI
Merge + DAPI
C
cleavage site
ss_pred: chhhHHHHHHHHHHHHHHHHHHHHH
Spitz: EKASIASGAMCALVFMLFVCLAFY
consensus: EtASIAgGatlAv~l~vi~c~~~y
.+|.+++-+++.=+.++++.+.+-
consensus: ~~AA~asT~ImVPvglvFvvFAvH
Orai1: EAAAIASTAIMVPCGLVFIVFAVH
ss_pred: hHHHHHHHHHHHHHHHHHHHHHHHH
confidence: 357777666655555555544433
D
TMD4
extracellular
cytoplasm
hOrai1 232 GQAAAIASTTIMVPFGLIFIVFAVHFYRSL 261
mOrai1 235 GEAAAIASTAIMVPCGLVFIVFAVHFYRSL 264
* ******* **** **.************
E
mOrai1 TM4 WT
mOrai1 A237F
mOrai1 A238F
mOrai1 A239F
mOrai1 I240F
mOrai1 A241F
mOrai1 S242F
mOrai1 T243F
mOrai1 A244F
mOrai1 I245F
mOrai1 237-239F
mOrai1 240-242F
mOrai1 243-245F
mOrai1 237-245F (F9)
pCDNA
RHBDL2
RHBDL2 SA
0.1
0.2
0.3

Figure S1, related to Figure 1

Characterisation of RHBDL2 cleavage of Orai1 TMD4. **A**. HEK293 cells were transfected with pcDNA, 3xHA-RHBDL2 or RHBDL2 SA, and indicated AP-TMDs for 48 hours. Released AP was collected over the final 16 hours. Values represent the level of released alkaline phosphatase/total alkaline phosphatase. n = 2 biological repeats for each AP-TMD. Error bars represent SEM. **B**. Immunofluorescent labelling of HA (green) and myc (red) epitopes in HEK293 cells transfected with hOrai1-3xmyc and 3xHA-RHBDL2 24 hours prior to fixation. Nuclear DNA is labelled with DAPI. Black boxes indicate that this channel was not imaged. Scale bars = 10 µm. Note that internal Orai1 positive structures are only observed upon co-expression with RHBDL2. **C**. HHpred alignment of the transmembrane domain of *Drosophila* Spitz with that of TMD4 in mouse Orai1. ss_pred and confidence is the PSI-PRED secondary structure prediction and confidence values, indicating alpha-helical structure (*h = helix, c= unstructured*). The consensus line indicates the profile that was generated for the target and the query proteins. The fourth line indicates which residues align and their similarity ("I" = very good, "+" = good, "." = neutral and "=" = clash). Residue colours: blue = acidic, red = basic, green = hydrophobic, black = polar/neutral. **D**. MSA of human and mouse Orai1 TMD4 sequence, depicting the respective conservation, numbering and boundaries of the TMD regions. **E**. Cells treated as in A, but here the values have been converted into a heat-map.

Figure S2

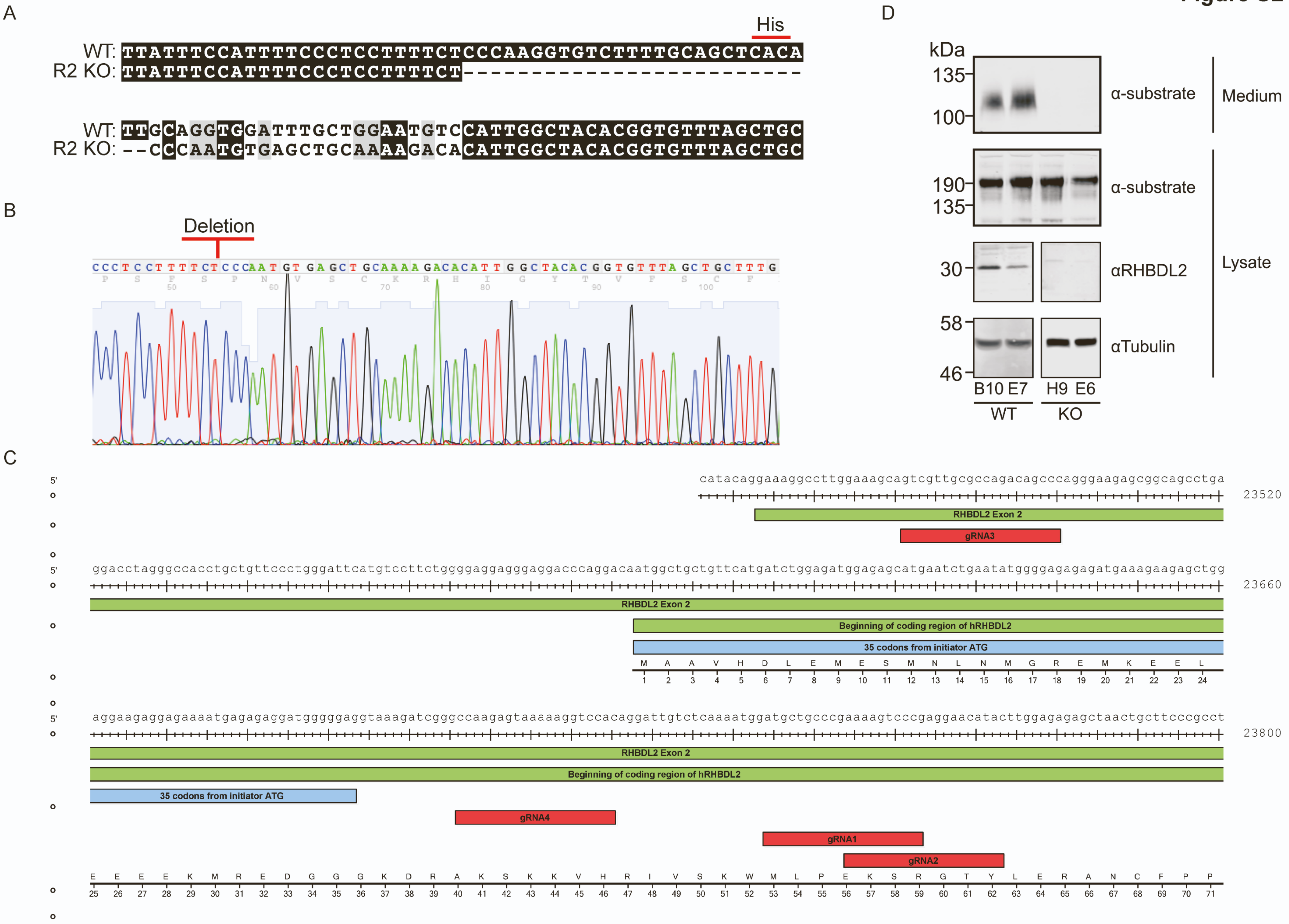

A
His
WT: TTATTTCCATTTTCCCTCCTTTTCTCCCAAGGTGTCTTTTGCAGCTCACA
R2 KO: TTATTTCCATTTTCCCTCCTTTTCT-------------------------
WT: TTGCAGGTGGATTTGCTGGAATGTCCATTGGCTACACGGTGTTTAGCTGC
R2 KO: --CCCAATGTGAGCTGCAAAAGACACATTGGCTACACGGTGTTTAGCTGC
B
Deletion
C
5'
catacaggaaaggccttggaaagcagtcgttgcgccagacagcccagggaagagcggcagcctga
23520
RHBDL2 Exon 2
gRNA3
5'
ggacctagggccacctgctgttccctgggattcatgtccttctggggaggagggaggacccaggacaatggctgctgttcatgatctggagatggagagcatgaatctgaatatggggagagagatgaaagaagagctgg
23660
RHBDL2 Exon 2
Beginning of coding region of hRHBDL2
35 codons from initiator ATG
M A A V H D L E M E S M N L N M G R E M K E E L
1 2 3 4 5 6 7 8 9 10 11 12 13 14 15 16 17 18 19 20 21 22 23 24
5'
aggaagaggagaaaatgagagaggatgggggaggtaaagatcgggccaagagtaaaaaggtccacaggattgtctcaaaatggatgctgcccgaaaagtcccgaggaacatacttggagagagctaactgcttcccgcct
23800
RHBDL2 Exon 2
Beginning of coding region of hRHBDL2
35 codons from initiator ATG
gRNA4
gRNA1
gRNA2
E E E E K M R E D G G G K D R A K S K K V H R I V S K W M L P E K S R G T Y L E R A N C F P P
25 26 27 28 29 30 31 32 33 34 35 36 37 38 39 40 41 42 43 44 45 46 47 48 49 50 51 52 53 54 55 56 57 58 59 60 61 62 63 64 65 66 67 68 69 70 71
D
kDa
135
100
α-substrate
Medium
190
135
α-substrate
30
αRHBDL2
Lysate
58
46
αTubulin
B10 E7
H9 E6
WT
KO

Figure S2, related to STAR Methods

Generation of a RHBDL2 null cell lines. **A-B**. Sanger sequencing reads and alignments from WT HEK293 and R2 KO cells, showing the nucleotides flanking the catalytic histidine (CAC = His) in RHBDL2. **C.** CRISPR/Cas9 targetting scheme for RHBDL2 in HaCaT cells. **D.** Western blot for RHBDL2, and its protease activity against a confirmed shed substrate, in wild type HaCaT clones B10 and E7 and knock-out (KO) clones E6 and H9. In Figure 6C-F, only B10 and H9 clones were used.

Figure S3

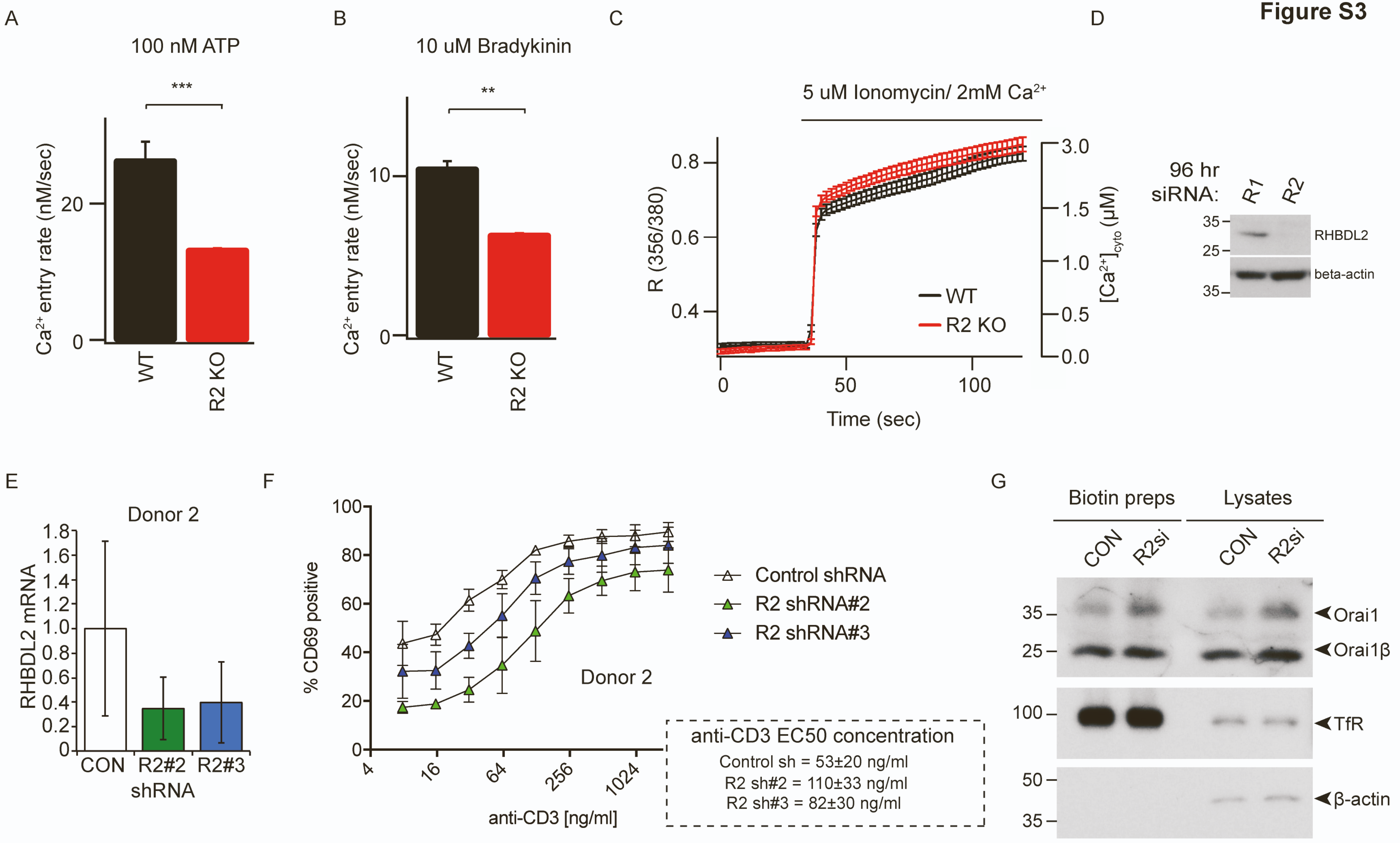

A
100 nM ATP
***
Ca2+ entry rate (nM/sec)
WT
R2 KO
B
10 uM Bradykinin
**
Ca2+ entry rate (nM/sec)
WT
R2 KO
C
5 uM Ionomycin/ 2mM Ca2+
R (356/380)
[Ca2+]cyto (μM)
WT
R2 KO
Time (sec)
D
96 hr siRNA:
R1
R2
RHBDL2
beta-actin
E
Donor 2
RHBDL2 mRNA
CON
R2#2
R2#3
shRNA
F
% CD69 positive
Control shRNA
R2 shRNA#2
R2 shRNA#3
Donor 2
anti-CD3 [ng/ml]
anti-CD3 EC50 concentration
Control sh = 53±20 ng/ml
R2 sh#2 = 110±33 ng/ml
R2 sh#3 = 82±30 ng/ml
G
Biotin preps
Lysates
CON
R2si
CON
R2si
Orai1
Orai1β
TfR
β-actin

Figure S3, related to Figures 3, 5 and 5

Further analysis of RHBDL2 deficient cells. A-B. Aggregate data from cells treated as in Figure 3G-H are plotted, analysing the rate of Ca^{2+} entry after ATP (in A) or Bradykinin (in B) treatment. **C.** Cytosolic Fura-2 fluorescence in wild type and RHBDL2 mutant HEK293 cells stimulated with 5 μM ionomycin in 2mM Ca^{2+} containing buffer. NB: as the calcium concentration exceeded the estimated limits for Fura-2 calibration in terms of μM Ca^{2+}, we have included both the calibrated values and the R values for this experiment. **D.** Western blots of HaCaT keratinocytes treated with RHBDL1 or RHBDL2 siRNAs for 96 hours, probed with RHBDL2 and beta-actin antibodies. **E**. TaqMan assays for RHBDL2 mRNA levels in T cells transduced with virus encoding control or RHBDL2 shRNAs. Error bars represent RQ standard error. **F.** T cell activation was measured by quantification of surface CD69 expression by FACS. CD69 expression is compared between control and RHBDL2 shRNA transduced primary CD4-positive T cells after stimulation with varying doses of platebound CD3. Each trace represents three biological replicates. In the dashed box, the calculated EC50 of anti-CD3 for each shRNA condition is indicated. Error indicates SEM. **G**. Western blots of HaCaT lysates and cell surface biotinylation preps after cells were treated with control or RHBDL2 siRNAs for 72 hours, labelled for endogenous Orai1, Transferrin receptor and beta-actin. Full length Orai1 (FL) and Orai1β (FLβ) are indicated by arrowheads. Orai1β arises from alternative start sites methionine-64 or -71 (Fukushima et al., 2012).

Figure S4

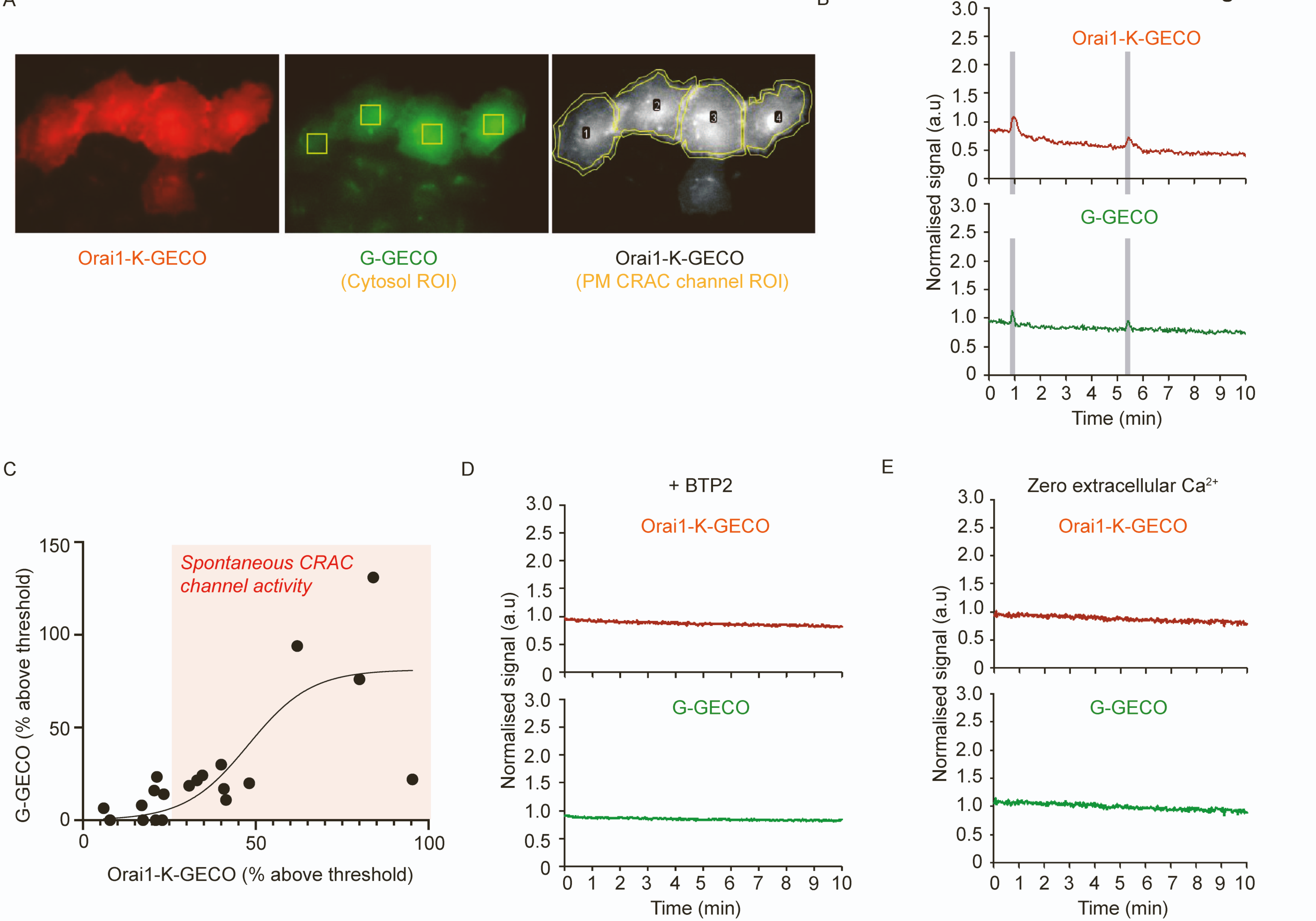
A
Orai1-K-GECO
G-GECO
(Cytosol ROI)
Orai1-K-GECO
(PM CRAC channel ROI)
B
Orai1-K-GECO
G-GECO
Normalised signal (a.u)
Time (min)
C
Spontaneous CRAC channel activity
G-GECO (% above threshold)
Orai1-K-GECO (% above threshold)
D
+ BTP2
Orai1-K-GECO
G-GECO
Normalised signal (a.u)
Time (min)
E
Zero extracellular Ca^{2+}
Orai1-K-GECO
G-GECO
Normalised signal (a.u)
Time (min)

Figure S4, related to Figure 6

Development of a spontaneous CRAC channel activity reporter assay. **A**. Snapshots of fluorescence imaging of live HaCaT cells expressing Orai1-K-GECO or G-GECO, with indicated regions of interest (ROIs) from which readings are collected. **B, D, E.** Example fluorescence line traces of HaCaT cells as in A, demonstrating spikes in Orai1-K-GECO that correlate with increased cytosolic G-GECO fluorescence (highlighted by grey lines). Importantly, no spikes in Orai1-K-GECO or G-GECO fluorescence are observed when cells are treated with the CRAC channel inhibitor BTP2 (in D), or are bathed in Ca^{2+} free media (in E). **C.** Correlation of Orai1-K-GECO fluorescence and cytosolic G-GECO fluorescence indicate that an >20% increase in Orai1-K-GECO fluorescence consistently raises cytosolic Ca^{2+} levels, which we define as spontaneous CRAC channel activity (highlighted by the pink box).

Figure S5

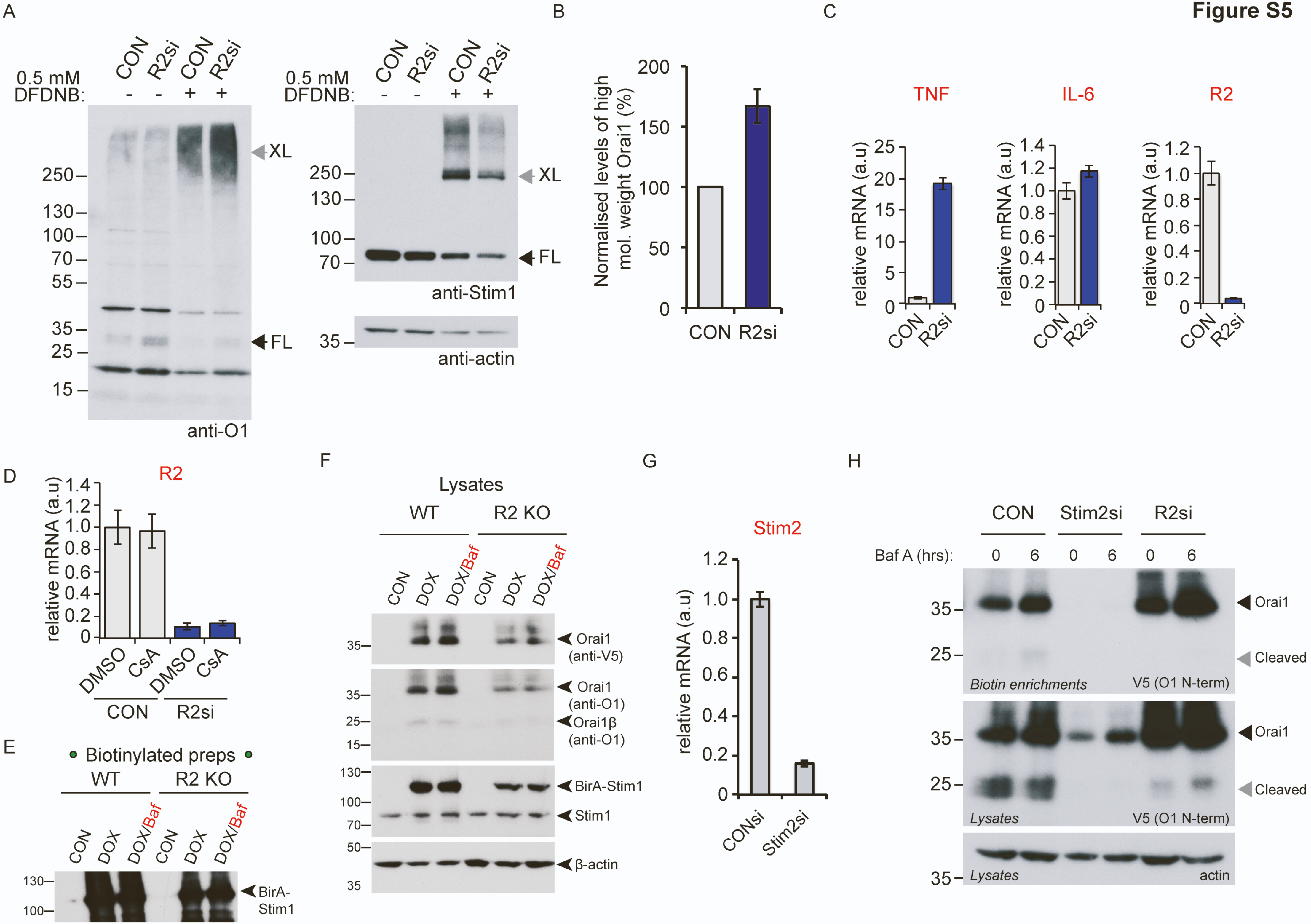

A
0.5 mM DFDNB: - - + +
CON R2si CON R2si
250
130
100
70
55
35
25
15
XL
FL
anti-O1
anti-Stim1
anti-actin
B
Normalised levels of high mol. weight Orai1 (%)
200
150
100
50
0
CON R2si
C
TNF
IL-6
R2
relative mRNA (a.u)
CON R2si
D
R2
relative mRNA (a.u)
DMSO CsA DMSO CsA
CON R2si
E
Biotinylated preps
WT R2 KO
CON DOX DOX/Baf CON DOX DOX/Baf
130
100
BirA-Stim1
F
Lysates
WT R2 KO
CON DOX DOX/Baf CON DOX DOX/Baf
Orai1 (anti-V5)
Orai1 (anti-O1)
Orai1β (anti-O1)
BirA-Stim1
Stim1
β-actin
G
Stim2
relative mRNA (a.u)
CONsi Stim2si
H
CON Stim2si R2si
Baf A (hrs): 0 6 0 6 0 6
Orai1
Cleaved
Biotin enrichments
V5 (O1 N-term)
Lysates
actin

Figure S5, related to Figure 6

Further analysis of the effect of RHBDL2 loss on Orai1. A. Western blots of HaCaT lysates after cells were treated with control or RHBDL2 siRNA for 96 hours, labelled for endogenous Orai1, Stim1 and beta-actin. Full length Orai1 (FL) and crosslinked Orai1 (XL) are indicated by arrowheads. Prior to lysis, cells were treated with 0.5 mM DFDNB for 1 hour, and subsequently quenched. **B**. Quantification of the fold change in Orai1 XL, from two independent experiments performed as in A. Error bars represent the SEM. **C-D.** TaqMan assay for TNF alpha, IL-6 and RHBDL2 mRNA levels in HaCaT cells treated with control or RHBDL2 siRNAs for 48 hours (in C) or 72 hours (in D). In D, Cyclosporin A (1 µm) was added for the final 24 hours. Error bars represent RQ standard error. Each bar represents one of at least four biological replicates. **E-F**. Western blots on biotinylated preparations and lysates from WT and RHBDL2 KO HaCaT cells, probed with indicated antibodies and relating to experiments in Figure 6F. **G.** Representative TaqMan assay for Stim2 mRNA levels in HaCaT cells treated with control or Stim2 siRNAs for 72 hours. **H.** Western blots of neutravidin agarose-based biotin captured lysates from HaCaT cells treated with Stim2 or RHBDL2 siRNAs for 96 hours. The expression of V5-Orai1 and Stim1-BirA* was induced with doxycycline (DOX, 250 µg/ml final) for 96 hours in the presence of 50 µm biotin. Six hours prior to lysis, bafilomycin A1 (BAF, 100 nm final) was added to block lysosomal degradation. Blots were probed for the N-terminal Orai1 V5 epitope. N-terminal cleavage products are indicated by the grey arrowheads.

Figure S6

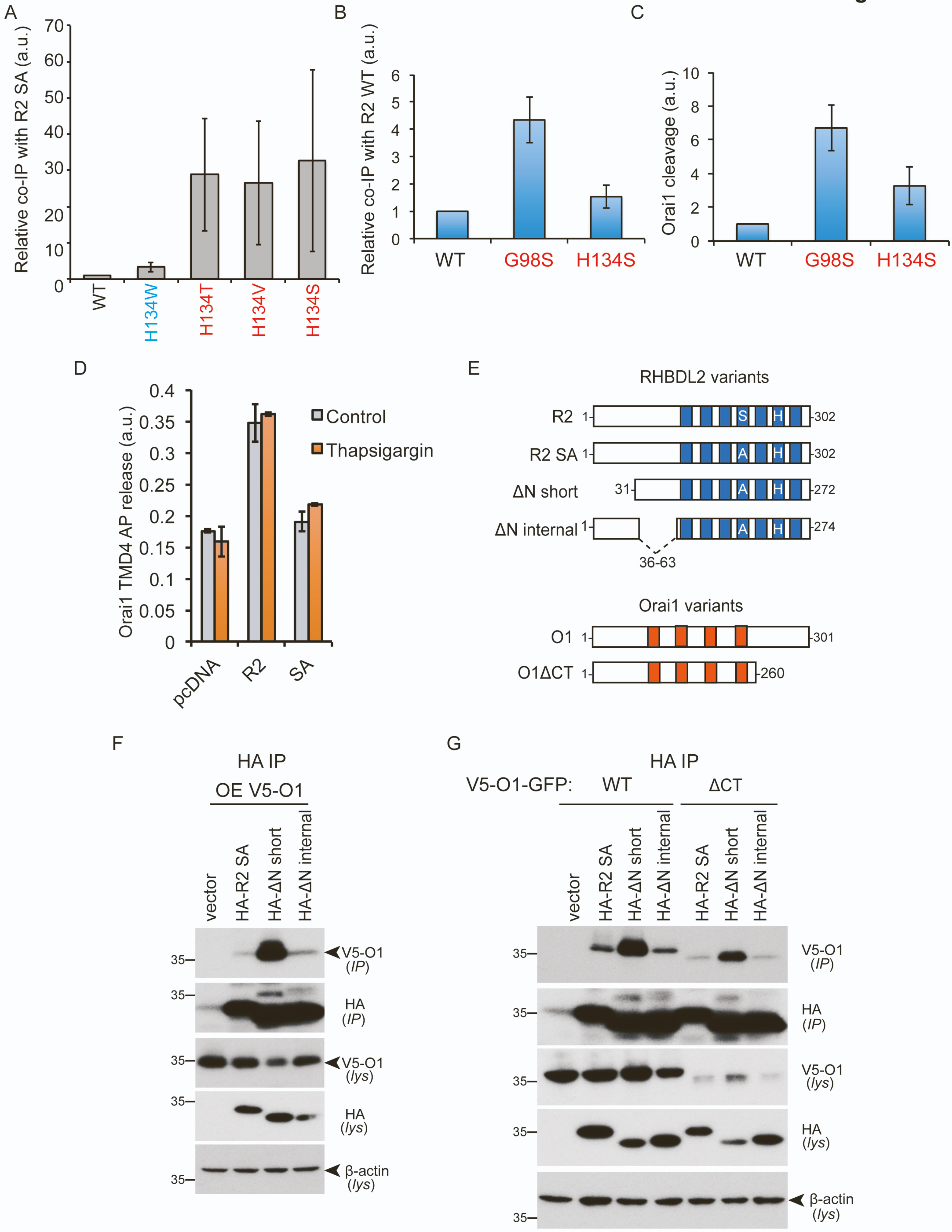

A
Relative co-IP with R2 SA (a.u.)
0
10
20
30
40
50
60
70
WT
H134W
H134T
H134V
H134S
B
Relative co-IP with R2 WT (a.u.)
0
1
2
3
4
5
6
WT
G98S
H134S
C
Orai1 cleavage (a.u.)
0
2
4
6
8
10
WT
G98S
H134S
D
Orai1 TMD4 AP release (a.u.)
0
0.05
0.1
0.15
0.2
0.25
0.3
0.35
0.4
Control
Thapsigargin
pcDNA
R2
SA
E
RHBDL2 variants
R2 1 S H 302
R2 SA 1 A H 302
ΔN short 31 A H 272
ΔN internal 1 A H 274
36-63
Orai1 variants
O1 1 301
O1ΔCT 1 260
F
HA IP
OE V5-O1
vector
HA-R2 SA
HA-ΔN short
HA-ΔN internal
35
V5-O1 (IP)
HA (IP)
V5-O1 (lys)
HA (lys)
β-actin (lys)
G
HA IP
V5-O1-GFP:
WT
ΔCT
vector
HA-R2 SA
HA-ΔN short
HA-ΔN internal
HA-R2 SA
HA-ΔN short
HA-ΔN internal
35
V5-O1 (IP)
HA (IP)
V5-O1 (lys)
HA (lys)
β-actin (lys)

Figure S6, related to Figure 7

Further analysis of the RHBDL2-Orai1 interaction. **A-C**. Quantification of RHBDL2-Orai1 binding (in A-B), or Orai1 cleavage product production (in C) from biological replicates of experiments described in Figure 7A-C. **D**. HEK293 cells were transfected with pcDNA, 3xHA-RHBDL2 or RHBDL2 SA, and AP-TMD4 from Orai1 for 48 hours. Cells were then either treated without or with thapsigargin for 30 mins. Total released AP was collected over the final 16 hours, including the time of thapsigargin treatment. Values represent the level of released alkaline phosphatase/total alkaline phosphatase. Error bars represent SEM**. E.** Domain diagrams depicting the mutant forms of RHBDL2 and Orai1 used in Figure S8F-G. **F-G**. HA immunoprecipitates (IP) and inputs from HEK293 cells transiently expressing full length or deletion mutant forms of 3xHA-RHBDL2 SA and wild type or mutant V5-Orai1-GFP for 24 hours were immunoblotted for V5, HA and beta-actin.

Figure S7

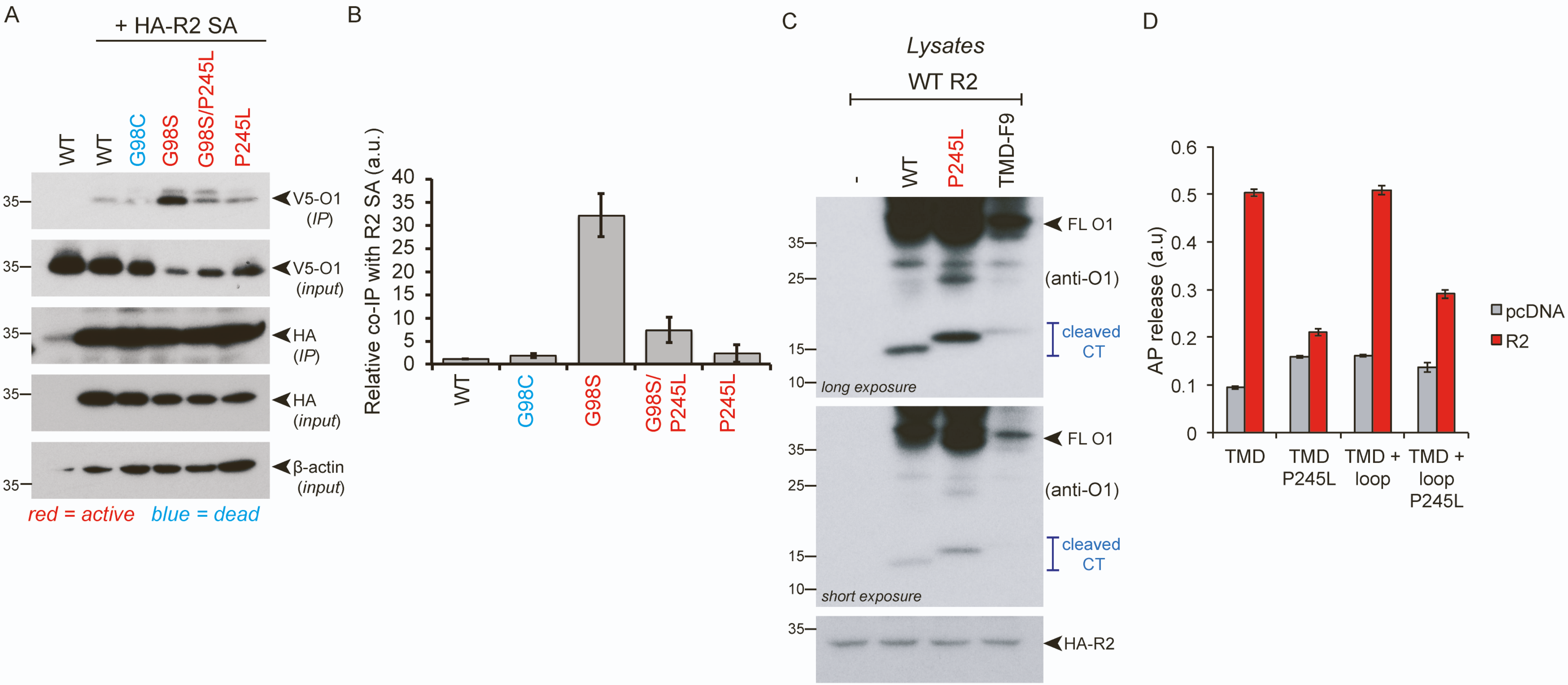

A
+ HA-R2 SA
WT
WT
G98C
G98S
G98S/P245L
P245L
35
V5-O1 (IP)
V5-O1 (input)
HA (IP)
HA (input)
β-actin (input)
red = active
blue = dead
B
Relative co-IP with R2 SA (a.u.)
40
35
30
25
20
15
10
5
0
WT
G98C
G98S
G98S/ P245L
P245L
C
Lysates
WT R2
-
WT
P245L
TMD-F9
35
25
15
10
FL O1
(anti-O1)
cleaved CT
long exposure
short exposure
HA-R2
D
AP release (a.u)
0.6
0.5
0.4
0.3
0.2
0.1
0
TMD
TMD P245L
TMD + loop
TMD + loop P245L
pcDNA
R2

Figure S7, related to Figure 7

Effect of the P245L mutation on RHBDL2 recognition and processing of Orai1. **A.** HA immunoprecipitates (IP) and inputs from HEK293 cells transiently expressing 3xHA-RHBDL2 SA and wild type or mutant V5-Orai1 for 24 hours were immunoblotted for V5, HA and beta-actin. **B.** Quantification of three biological replicates of the experiment performed in A**.** **C.** Lysates from HEK293 cells transiently expressing 3xHA-RHBDL2 WT and wild type or mutant forms of V5-Orai1 (either P245L, or a systematic mutation of all 9 extracellular amino-acids in TMD4 to phenylalanine, termed “F9”) for 48 hours were immunoblotted for O1 and HA. **D.** HEK293 cells were transfected with pcDNA or 3xHA-RHBDL2, and AP-TMD4, AP-TMD4 fused to the extracellular loop that connects TMD3 and TMD4 (TMD+loop), either as in wild type or as a mutant equivalent to P245L in hOrai1, for 48 hours. Total released AP was collected over the final 16 hours. Values represent the level of released alkaline phosphatase/total alkaline phosphatase. Error bars represent SEM**.**
